# Metatranscriptomic Analysis of Corals Inoculated With Tolerant and Non-Tolerant Symbiont Exposed to High Temperature and Light Stress

**DOI:** 10.3389/fphys.2022.806171

**Published:** 2022-04-11

**Authors:** Ikuko Yuyama, Tomihiko Higuchi, Takuma Mezaki, Hisako Tashiro, Kazuho Ikeo

**Affiliations:** ^1^Graduate School of Science and Technology for Innovation, Yamaguchi University, Yamaguchi, Japan; ^2^Atmosphere and Ocean Research Institute, The University of Tokyo, Kashiwa, Japan; ^3^Kuroshio Biological Research Foundation, Otsuki, Japan; ^4^Department of Genomics and Evolutionary Biology, National Institute of Genetics, Mishima, Japan

**Keywords:** coral bleaching, endosymbiosis, *Cladocopium*, *Durusdinium*, *Acropora solitaryensis*, RNA-seq

## Abstract

Algal symbionts of corals can influence host stress resistance; for example, in the Pacific Ocean, whereas *Cladocopium* (C-type) is generally dominant in corals, *Durusdinium* (D-type) is found in more heat-resistant corals. Thus, the presence of D-type symbiont likely increases coral heat tolerance, and this symbiotic relationship potentially provides a hint to increase the stress tolerance of coral–algal symbioses. In this study, transcriptome profiles of *Cladocopium-* and *Durusdinium*-harboring *Acropora solitaryensis* (C-coral and D-coral, respectively) and algal photosystem functioning (*F*_*v*_*/F*_*m*_) under bleaching conditions (high temperature and light stress) were compared. Stress treatment caused algal photoinhibition that the *F*_*v*_/*F*_*m*_ value of Symbiodiniaceae was immediately reduced. The transcriptome analysis of corals revealed that genes involved in the following processes were detected: endoplasmic reticulum (ER) stress, mitophagy, apoptosis, endocytosis, metabolic processes (acetyl-CoA, chitin metabolic processes, etc.), and the PI3K-AKT pathway were upregulated, while DNA replication and the calcium signaling pathway were downregulated in both C- and D-corals. These results suggest that unrepaired DNA and protein damages were accumulated in corals under high temperature and light stress. Additionally, some differentially expressed genes (DEGs) were specific to C- or D-corals, which includes genes involved in transient receptor potential (TRP) channels and vitamin B metabolic processes. Algal transcriptome analysis showed the increased expression of gene encoding photosystem and molecular chaperone especially in D-type symbiont. The transcriptome data imply a possible difference in the stress reactions on C-type and D-type symbionts. The results reveal the basic process of coral heat/light stress response and symbiont-type-specific coral transcriptional responses, which provides a perspective on the mechanisms that cause differences in coral stress tolerance.

## Introduction

Mass coral bleaching, which mainly occurs due to elevated sea surface temperatures, is a state in which algal symbionts (family Symbiodiniaceae, hereinafter referred to as “symbionts”) are decreased in host corals over a wide area. Bleaching phenomena cause fatal impact in the most cases to host corals because the loss of alga does not provide the expected amount of energy source to the host (Baker et al., 2008; Baird et al., 2017). Coral bleaching, caused by high temperatures and strong irradiance, is accompanied by photoinhibition of Symbiodiniaceae (Lesser and Shick, 1989; Warner et al., 1999; Gorbunov et al., 2001; Lesser, 2011). Photoinhibition refers to light-induced reduction of the photosynthetic rate as a result of the generation of reactive oxygen species (ROS) from the excess light energy over the limits of the CO_2_ fixation process (Murata et al., 2007). ROS damage the reaction center of photosystem II (PSII), and high temperatures inhibit the repair of this damage, which results in a decline of the photosynthetic rate of symbiont algae (Takahashi et al., 2009). Symbiont-produced ROS can also damage lipids, proteins, and DNA of host corals and increase expression and/or activity of the antioxidant enzymes superoxide dismutase and catalase in corals (Lesser, 1997; Higuchi et al., 2009). In addition, the generation of nitric oxide (NO) has also been reported as a key factor of bleaching phenomena (Trapido-Rosenthal et al., 2005; Perez and Weis, 2006; Bouchard and Yamasaki, 2008; Hawkins et al., 2013).

The underlying process of coral bleaching has been studied at the molecular level and the expression changes of heat shock proteins (Hsp, DnaJ) (Császár et al., 2009; Meyer et al., 2011; Yuyama et al., 2012; Maor-Landaw and Levy, 2016), antioxidant enzymes (catalase, thioredoxin, MnSOD) (Edge et al., 2005; Császár et al., 2009; Seneca et al., 2010; Maor-Landaw and Levy, 2016), immune system-related genes (TNF receptors, TNF receptor-associated factors) (Voolstra et al., 2009; Barshis et al., 2013), and metabolic process-related genes (carbonic anhydrase, adenine transporter, and calcium channels) (Edge et al., 2005; Meyer et al., 2011) have been reported. Furthermore, the involvement of apoptosis and autophagy-related proteins, such as Bcl-2 and caspase, in the bleaching response was also revealed by the studies using inhibitors and by investigating their gene expression patterns (Dunn et al., 2007; Kvitt et al., 2016). The recent large-scale transcriptome analyses using *Exaiptasia* and *Acropora hyacinthus* have highlighted ER stress as an associated pathway involved in bleaching phenomena (Oakley et al., 2017; Ruiz-Jones and Palumbi, 2017). With the development of next-generation sequencing analysis, the number of reports on the molecular response to bleaching is increasing. Transcriptome analysis *in hospite* Symbiodiniaceae has also been carried out, and light-harvesting complex (LHC), heat-shock, and photosystem-constitutional proteins have been reported as the heat stress-responsive transcripts (Rosic et al., 2011; Gierz et al., 2017).

Although symbiont type has a large effect on the bleaching sensitivity of corals, less is known about how different symbiont types contribute to coral stress tolerance. There are seven genera in Symbiodiniaceae, among which *Cladocopium* is ubiquitous genus associated with corals, while *Durusdinium* is associated with high-temperature stress tolerance in corals. It has been reported that during a bleaching event, the dominant symbiont type in the coral shifts from the general symbiont *Cladocopium* (previously classified as *Symbiodinium* clade C, hereafter referred to as C-type) to a stress-resistant type, *Durusdinium* (previously *Symbiodinium* clade D, hereafter referred to as D-type) (Baker, 2003; Baker et al., 2004; Berkelmans and van Oppen, 2006; LaJeunesse et al., 2018). The influence of each symbiont on the stress sensitivity of corals has been revealed by genotyping the symbionts of corals that survive a bleaching event (Baker, 2003; Berkelmans and van Oppen, 2006) and by the physiological studies using corals associated with each symbiont type (Rowan, 2004; Abrego et al., 2008; Mieog et al., 2009; Yuyama and Higuchi, 2014; Yuyama et al., 2016). D-type colonized corals (hereafter referred to as D-corals) have a higher survival ratio under temperature stress than C-type-colonized corals (hereafter referred to as D-corals). At higher temperatures, the C-type shows a more pronounced photoinhibitory response, which negatively impacts coral viability (Mieog et al., 2009; Yuyama et al., 2016). The C-type has a higher carbon fixation rate than does the D-type, and it promotes coral growth to a greater extent (Cantin et al., 2009). The efficiency of nitrogen acquisition also differs between C- and D-types; the C-type has a higher acquisition rate at normal temperatures, but a lower rate than that of the D-type at higher temperatures (Baker et al., 2013). Thus, the contributions of the D-type and C-type to coral growth and nutrient sources vary with the temperature (Cunning et al., 2015).

However, there are knowledge gaps in the difference in bleaching sensitivity derived from each symbiont due to the difficulty of preparing corals associated with symbionts, although there are a few molecular studies recently reported (Cunning and Baker, 2020; Rodriguez-Casariego et al., 2022). To address this gap, we first attempted to prepare a model symbiosis system suitable for gene expression analysis, namely, juvenile corals harboring cultured monoclonal C-type or D-type Symbiodiniaceae (Yuyama and Higuchi, 2014; Yuyama et al., 2018). The advantages of using such juvenile corals are as follows: (1) the prevention of contamination by other organisms, because juvenile corals can be kept in filtered seawater or artificial seawater, (2) selected alga can be introduced, and (3) a more homogenous response can be detected (Yuyama et al., 2005) (adult colonies have large variations in physiological responses). To clarify the different influences of C- and D-type symbionts on coral bleaching as well as a common molecular mechanism of coral bleaching, we exposed the coral associated with each symbiont to high temperature and light stress. Then, the maximum quantum yield of PSII (*F*_*v*_*/F*_*m*_) of symbiont was measured by pulse amplitude-modulated (PAM) fluorometry and performed large-scale gene expression analysis of coral–algal associations.

## Materials and Methods

### Symbiodiniaceae and Coral Samples

**Figure 1:**
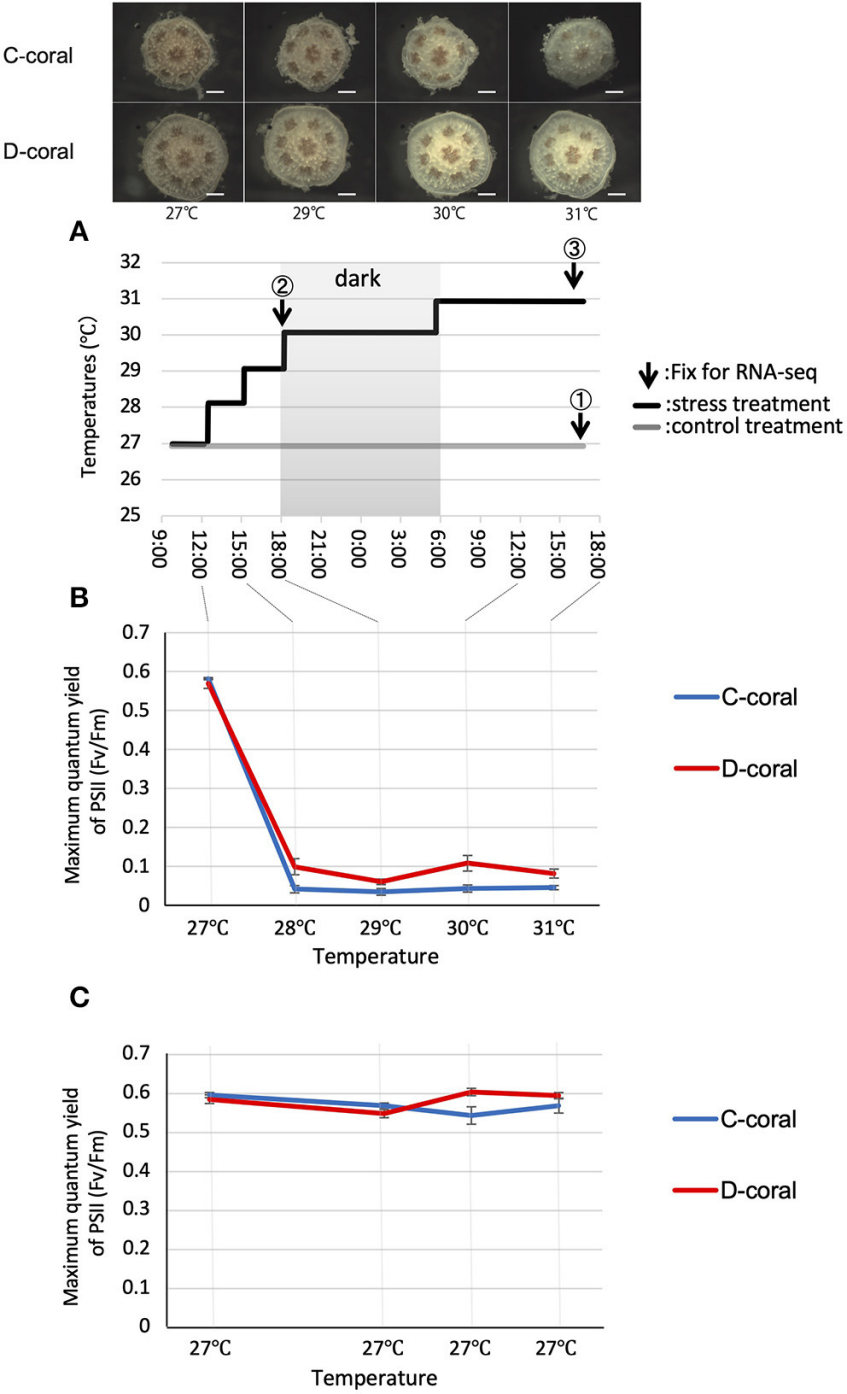
**(A)** Experimental design for stress experiments and stereoscopic microscope images of *Acropora solitaryensis* during temperature increase from 27 to 31°C. The upper pictures show *A*. *solitaryensis* colonized by C-type algae (C-coral); lower pictures show *A*. *solitaryensis* colonized by D-type algae (D-coral). The graph shows the transition of the temperature setting during the experiment. Arrows indicate the time of fixation for RNA-seq analysis. The samples derived from each time point are referred to as day 1 (at 29°C, collected before setting 30°C), day 2 (at 31°C), and control. For RNA-seq analysis, we prepared two replicates (each replicate with 15 pooled colonies) for each treatment (stress conditions and control conditions) (Supplementary Figure S2). Scale bars = 0.5 mm. **(B,C)** Maximum quantum yield (*Fv/Fm*) of C-type (*Cladocopium*) and D-type (*Durusdinium*) Symbiodiniaceae associated with corals in stress **(B)** and control treatments **(C)**. No significant difference was found between C-type and D-type in same treatment; however, there were significant differences between control and stress treatments in both C-type and D-type (Turkey–Kramer HSD, *p* < 0.05). Values are means ± SE.

Symbiodiniaceae strains CCMP 2556 (D-type, genus *Durusdinium*) and CCMP 2466 (C-type, genus *Cladocopium*) were purchased from the Bigelow Laboratory for Ocean Sciences (West Boothbay Harbor, ME, USA; https://ccmp.bigelow.org). *Acropora solitaryensis* larvae were generated from ~5 different colonies located in the area of 32°48′16.5″ N 132°39′11.1″ E (permit number 106, Kochi Prefecture) as described by Omori and Iwao (Omori and Iwao, 2014). Planula larvae were induced to metamorphose into polyps with neuropeptide Hym248 (Iwao et al., 2002) and infected with the two Symbiodiniaceae alga as described by Yuyama et al. (2016, 2018). Each Symbiodiniaceae strain (~1,000 cells per polyp) was introduced to corals 1 week after metamorphosis; polyps were grown in petri dishes (55 mm) at 26–27°C with a 12-h light–dark cycle at 80 μmol m^−2^ s^−1^. As C-type algae take ~3 months to colonize corals, corals maintained for 3 months were used in the stress experiments (Yuyama and Higuchi, 2014; Yuyama et al., 2016) (Figure 1, Supplementary Figure S1). To verify the genotypes of Symbiodiniaceae colonizing corals, Restriction Fragment Length Polymorphism (RFLP) analysis was performed with five corals, each, as described in Yuyama and Higuchi (2014) (Supplementary Figure S3). Corals were maintained without feeding. During this 3-month incubation period, the corals associated with D-type (D-corals) tended to be larger than those associated with C-type (C-corals) (data not shown), as previously reported in *Acropora tenuis* (Yuyama and Higuchi, 2014).

### Stress (High Temperature and Strong Light) Exposure Experiments

A number of two separate experiments were performed using the same conditions to avoid the effects of photography and FvFm measurements on RNA-seq samples (Supplementary Figure S2). First, each five C- and D-corals were incubated under bleaching conditions (high temperature and strong light) to monitor the coral bleaching states including photosynthetic responses of the endosymbiotic algae. The corals were incubated under a 12-h light–dark cycle at high irradiance (1,500 μmol m^−2^ s^−1^) and the water temperature was increased gradually from 27 to 31°C over 21 h. The temperature was controlled by a thermo-controller (TC-101; Eaton, Tokyo, Japan). We confirmed that the temperature accuracy was within ± 0.5°C by thermometer under digital microscope (VHX2000; Keyence, Osaka, Japan). This bleaching condition was determined in the preliminary experiments conducted with juvenile *A. solitaryensis* corals. Coral polyps were photographed with a stereomicroscope (LZ; Kenis, Osaka, Japan) mounted on a CMOS camera (Tucsen Photonics, Fuzhou, China). These photographs enabled us to confirm the symbiont cell densities in the coral tissue, and to visualize the decrease therein (Supplementary Figure S1). A number of two containers for the experimental control population (each with five corals associated with C-type or D-type) were maintained at 27°C under a 12-h light/dark cycle at 80–100 μmol m^−2^ s^−1^ (Figure 1).

Second, other corals were exposed to the same stress conditions described above and fixed after 6 (29°C) or 30 h (31°C) to prepare the samples for transcriptome analysis. Approximately 90 colonies each were prepared for C- and D-type corals as the samples for RNA-seq. Among them, 30 corals each were used for the control, stress day 1 and stress day 2 treatments. These 30 corals were divided into two containers and prepared as the two biological replicates. For the control condition for RNA-seq analysis, corals were maintained at 27°C and 80 μmol m^−2^ s^−1^ for ~2 months and then fixed at the same time as the stress day 2 corals. Due to the limited number of samples, the control coral for the second day of stress (stress day 2) treatment was also used as the control for the first day of stress (stress day 1) treatment. Here, it was assumed that the expression pattern would not change even if the fixation time for RNA-seq was shifted by 1 day, since the condition is stable after 2 months of culture. Corals were fixed on day 1 (after 6 h, at 29°C) and on day 2 (after 30 h at 31°C) in RNAlater (Ambion, Austin, TX, USA) and stored at −80°C. The control population was maintained at 27°C and 80 μmol m^−2^ s^−1^ (12-h light–dark cycle) and fixed after 30 h.

### Measurement of Photosynthetic Parameter (F_v_/F_m_)

The photosynthetic parameter (*F*_*v*_*/F*_*m*_) of endosymbiotic algae was monitored by PAM fluorometry (MiniPAM; Walz GmbH, Effeltrich, Germany) (Yuyama et al., 2016). All *F*_*v*_*/F*_*m*_ measurements were taken after a 15-min dark adaptation period. In the measurement, PAM probes were fixed near the coral body wall. *F*_*v*_*/F*_*m*_ values for the symbiotic C-type and D-type were compared for the different treatments. *Post-hoc* differences were assessed using the Tukey–Kramer honestly significant difference test (Anaconda 3-5.2.0, Python version 3.6.5; Continuum Analytics, Austin, TX, USA).

### Counting Algal Symbiont (Estimating Symbiont Density)

After the end of the stress experiment, corals were fixed in 3% formaldehyde to count symbiont cells inside corals (corals from the control and stress day 2 treatments were fixed to investigate the symbiont density). The fixed corals were decalcified in solution containing 0.5 M EDTA and homogenized in 0.01% Triton X using the method described by Yuyama and Higuchi (2014). Then, symbiont cells in the coral homogenate were counted using a hemocytometer (Thomas Scientific, Swedesboro, NJ, USA) under a digital microscope (VHX2000; Keyence, Osaka, Japan). To estimate symbiont density (cells/ mm^2^), the surface area of photographed corals was calculated using ImageJ software. These corals do not differ in height; thus, only the surface area was estimated. To clarify that a bleaching response was occurring, algal cell density ratios of “stress day 2” / “control” were calculated.

### Transcriptome Analysis

Each treatment was performed in two replicates, and a total of 12 RNA libraries were prepared. Total RNAs were extracted from each replicate using the PureLink RNA Mini Kit, and *Poly(A*) *RNA isolation* from total *RNA* was performed with a protocol of Next Poly(A) mRNA Magnetic Isolation Module (New England Biolabs, Ipswich, MA, USA). The RNA quality of each sample was checked using an Agilent 2100 Bioanalyzer (Agilent Technologies, Santa Clara, CA, USA) before producing the libraries. The sequencing libraries were generated from poly(A) mRNA using the NEBNext Ultra RNA Library Prep Kit (New England Biolabs). All libraries were sequenced on the HiSeq 4000 platform (Illumina, San Diego, CA, USA) by Macrogen Japan. The resulting 101-bp paired-end reads were first pre-processed [trimming of low-quality bases using a Phred quality score (Qv) <20 from the 5′ and 3′ ends of each read, and removing short reads (<25 bp) and low-quality reads (30% of bases with Qv ≤ 15)] using the DNA Data Bank of Japan (DDBJ) Read Annotation Pipeline (Nagasaki et al., 2013).

#### Transcriptome Analysis of Host Corals

Trimmed reads from all samples were assembled *de novo* using trinity in the DNA Data Bank of Japan (DDBJ) Read Annotation Pipeline (Nagasaki et al., 2013). Assembled contigs were translated using TransDecoder (Haas et al., 2013) to isolate likely coding sequences. The peptide sequences were filtered for redundancy using CD-Hit (v4.6.1; Fu et al., 2012) specifying a 95% similarity threshold and then obtained 168,491 contigs. To detect contig sequences originating from the host coral, we built custom coral and Symbiodiniaceae databases. The contigs derived from *A*. *solitaryensis* were isolated by the alignment to coral databases, and symbiont sequences were removed, as previously described (Yuyama et al., 2018). Reads were mapped to the *A*. *solitaryensis* contigs using the Bowtie2—very-sensitive algorithm (run with options: -D 20 -R 3 -N 0 -L 20 -i S,1,0.50) (Langmead and Salzberg, 2012). The eXpress was used to quantify the transcript abundances. To identify differentially expressed genes (DEGs) between the control and stress conditions (days 1 and 2), the edgeR method in the TCC package of R (Sun et al., 2013) was used (Supplementary Figure S2). Genes were determined to be significantly differentially expressed based on a false-discovery rate <0.05. All *A*. *solitaryensis* contigs were annotated using the public Swiss-Prot database and the non-redundant protein database (NCBI-nr) by BLASTx search with an e-value cutoff of 1e-4. Gene Ontology (GO) enrichment analyses were performed on the annotated dataset of DEGs using the Database for Annotation, Visualization, and Integrated Discovery (Huang et al., 2009b) (https://david.ncifcrf.gov/). In the analysis, annotated DEGs were compared with the annotation of the whole transcriptome, and GO terms enriched among the DEGs were identified. To summarize the major pathways involved in corals exposed to high-temperature/light, Kyoto Encyclopedia of Genes and Genomes (KEGG) pathway analyses (Kanehisa and Goto, 2000) of the DEGs detected in both C- and D-type corals were also performed. As the background for the GO enrichment analysis, the Swiss-Prot annotation results of all *A*. *solitaryensis* contigs were used.

#### Transcriptome Analysis of Algal Symbiont

Transcriptome analyses of Cladocopium and Durudsinium was performed using same RNA-seq short reads with the coral analysis. *De novo* assembly of C-type and D-type was performed, respectively, using FASTQ data of C- and D-type corals, obtained in this study. To detect coding sequences of the assembled contigs, TransDecoder (v. 2.0.1) was used (Haas et al., 2013). In total, 157,584 and 156,284 contigs were obtained from the C- and D-type coral datasets. Subsequently, contigs with more than 95% nucleotide sequence identity were removed using CD-HIT (v. 4.7; Fu et al., 2012). The contigs derived from C-type and D-type were isolated by alignment to coral databases and symbiont sequences were removed, as previously described (Yuyama et al., 2018). As a result, 98,707 and 97,948 contigs (clusters) were obtained from the C- and D-type corals (Supplementary Figure S3). The contigs derived from *Cladocopium and Durusdinium* were isolated by the alignment to custom Symbiodiniaceae databases and coral sequences using BLASTn (v. 2.2.30; NCBI, Bethesda, MD, USA). The Symbiodiniaceae database included the genomes of *Symbiodinium minutum* (Shoguchi et al., 2013), *Symbiodinium kawagutii* (Lin et al., 2015), *Symbiodinium tridacnidorum* and *Cladocopium* sp. (Shoguchi et al., 2018), and the transcriptomes of *Cladocopium* sp. and *Durusdinium* sp. (Ladner et al., 2012). The coral database included the *Acropora digitifera* genome (Shinzato et al., 2011) and transcriptomes of non-symbiotic *Acropora hyacinthus* (https://matzlab.weebly.com/data–code.html) and *A*. *tenuis* (Yuyama et al., 2018). Reads were mapped to *Cladocopium-* or *Durusdinium*-derived contigs with the Bowtie2 (v. 2.2.4) very-sensitive algorithm. Then, the quantification of transcript abundances and identification of DEGs were performed by the same method used for the identification of coral transcripts. The eXpress (v. 1.5.1) program was used to quantify transcript abundances and estimate fragments per kilobase of transcript per million mapped reads (FPKM) values (Roberts and Pachter, 2013). To identify transcripts expressed differentially between the control and stress conditions, analysis using iDEGES/edgeR in the TCC package (Sun et al., 2013) was performed. The differentially expressed transcriptome was annotated using Swiss-Prot and the non-redundant protein database. GO enrichment was also performed on the DAVID website (https://david.ncifcrf.gov/) (Huang et al., 2009a). In the analysis, all *Cladocopium* or *Durusdinium* transcripts annotated with GO terms were used as the background.

## Results

### Stress Response

During the increase in temperature, the appearances of both corals (C-coral and D-coral) remained largely unchanged until 6 h, and the color of the body gradually became white over the following 24 h (Figure 1, Supplementary Figure S4). To investigate the responses of endosymbiotic algae under high temperature and light stress, their photosynthetic efficiencies were measured (Figure 1). The photosynthetic ratios (*F*_*v*_*/F*_*m*_) of the C- and D-types were 0.582 ± 0.002 and 0.57 ± 0.013 (average ± standard error [SE]), at the beginning of the experiment; these values decreased to 0.042 ± 0.009 and 0.099 ± 0.002 (average ± SE), in the first 3 h. After this point, the photosynthetic activity of algae remained low until the end of the experiment. At 3 h, when the temperature was increased to 28°C under strong light, the D-type tended to show more photosynthetic activity than did the C-type. In the control treatment (27°C, 80 μmol m^−2^ s^−1^), no significant fluctuation of *F*_*v*_*/F*_*m*_ was observed in either type; the *F*_*v*_*/F*_*m*_ values were ~0.6. *F*_*v*_*/F*_*m*_ values were significantly decreased under stress compared with control conditions, in both C- and D-types (*p* < 0.05), although no significant difference was observed between the two types. At the end of the experiment, the corals were fixed and decalcified to measure the symbiont densities. Cell density tended to decrease during the experimental period, with a 51% decrease in C-type and a 28% decrease in D-type compared to control conditions (Supplementary Figure S4).

### Transcriptomic Change in Corals

For RNA-seq analysis, samples were fixed at 6 h (day 1, at 29°C) and 30 h (day 2, at 31°C) after the start of the experiment. We sequenced cDNA libraries derived from each sample, and ~40 million reads were obtained from each. Sequence data were deposited in the DDBJ Read Archive (DRA008078). As a result of the *de novo* assembly using reads from all samples, 663,795 contigs were generated. Subsequently, the protein-coding regions were estimated, and redundant sequences were removed, which resulted in 168,491 sequences. Among them, 40,036 contigs (DDBJ/ENA/GenBank accession codes ICPH01000001-ICPH01040036) assigned to the coral database by BLASTN search were used as a reference gene set of corals for subsequent analyses (Supplementary Tables S1, S2). All of the reads from each population were aligned to the reference using Bowtie2; we then attempted to detect DEGs in the two biological replicates. In the Bowtie2 analysis, ~14–16 million reads were mapped to reference contigs. The expression levels in the control population were compared with those in the stress day 1 or day 2 treatment in each coral, and DEGs under the stress conditions, as compared to controls, were detected. Of these DEGs, 9,537 were annotated by BLASTX searches against the UniProt/Swiss-Prot database. In the stress day 1 treatment, 1,685 and 1,081 DEGs were detected in C- and D-type corals, and in stress day 2, 2,954 DEGs and 3,516 DEGs were detected (Supplementary Figure S5).

**Figure 2:**
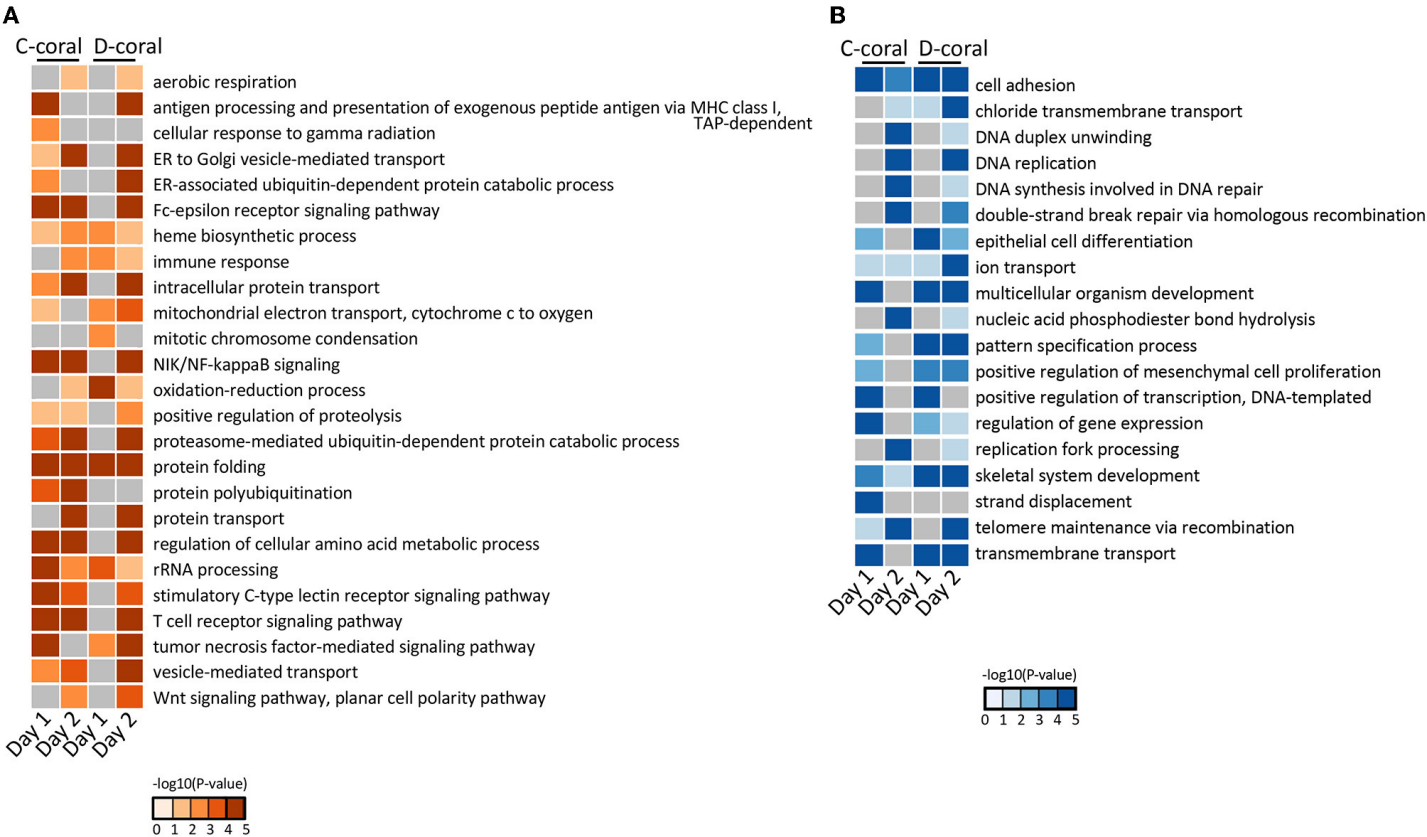
Heatmap of enrichment scores [–log10 (*p*-value)] from GO enrichment analysis using upregulated coral DEGs **(A)** and using downregulated coral DEGs **(B)**. The detected significantly (*p* < 0.05) enriched GO terms in biological process are shown.

Next, the UniProt accession IDs assigned to each DEG were used for GO enrichment analysis. DEGs from stress days 1 and 2 were subjected to GO enrichment analysis, and the top enriched GO terms with low *p*-values were selected and shown in Figure 2. GO terms related to the organs not found in cnidarians and GO terms with duplicate meanings were deleted. GO analysis revealed that the up-regulated genes are significantly enriched in protein folding, oxidation-reduction process, and immune response, whereas the down-regulated genes are highly involved in skeletal system development, DNA replication, and telomere maintenance *via* recombination (Figure 2). Their expression patterns indicated that the gene expression response of D-coral was slower than that of C-coral. Some metabolic systems, such as glucose, metabolic processes, and ammonium ion metabolic processes, were upregulated at 29°C (day 1) in C-corals, while they were hardly changed in D-corals; similar cases were also detected among downregulated genes.

**Figure 3:**
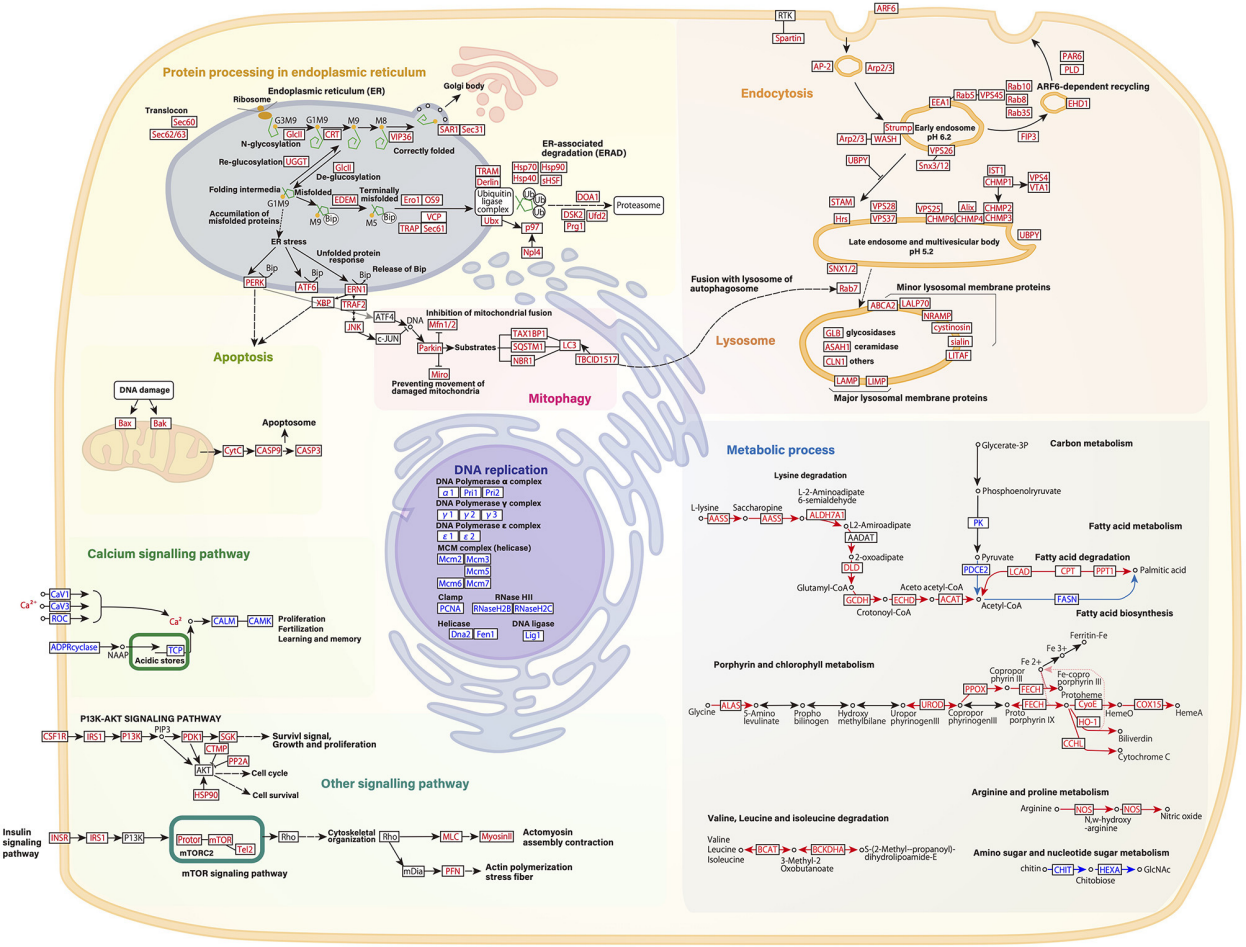
Schematic presentation of several gene pathways involved in high temperature and strong light stress based on RNA-seq data of host corals. Common changes in gene expression between C-coral and D-corals at 31 °C are indicated. Each pathway is described based on the KEGG pathway database (Kanehisa and Goto, 2000). Upregulated transcripts during high temperature and/light stress are shown in red; downregulated transcripts are indicated in blue. Each gene expression pattern is also shown in Supplementary Figure S1.

**Figure 4:**
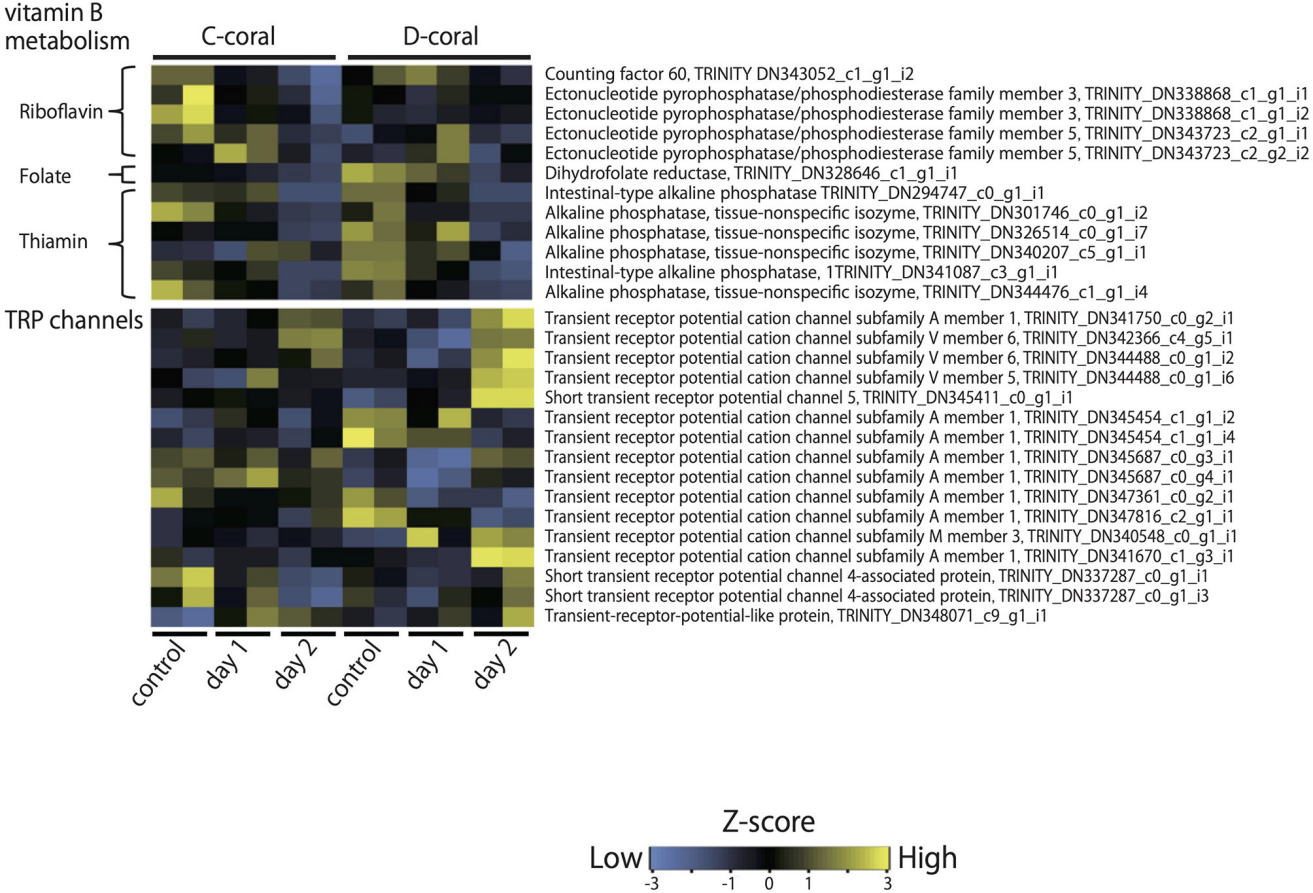
Heatmap displaying the relative expression values (*z*-score of TMM-normalized FPKM values) of contigs, which shows the different expression patterns of C- and D-corals during treatment with high temperature and strong light. As representative examples, the expression patterns of contigs encoding TRP channels and genes involved in vitamin B metabolism are shown. Expression values were converted into z-scores and plotted by heatmap.2 in R.

Kyoto Encyclopedia of Genes and Genomes pathway analysis was also performed to identify the major molecular processes among the DEGs. Transcriptome data at 31°C (day 2) were mainly used for KEGG pathway analysis. Figure 3 shows several upregulated genes, which include those involved in endocytosis, lysosome organization, acetyl-CoA, NO, chitin metabolic process, and the PI3K-AKT pathway, and downregulated genes, which include those involved in DNA replication and the calcium signaling pathway. In addition, specific DEGs for C- and D-type corals were detected from DEG gene list (Figure 4). As a result, inflammatory mediator regulation of transient receptor potential (TRP) channels was identified as a pathway specifically upregulated in D-type corals. Additionally, different types of vitamin B metabolic pathways were identified as downregulated under stress conditions in the two corals: riboflavin in C-coral, and folate and thiamine in D-coral.

### Transcriptomic Change in Algal Symbiont

**Figure 5:**
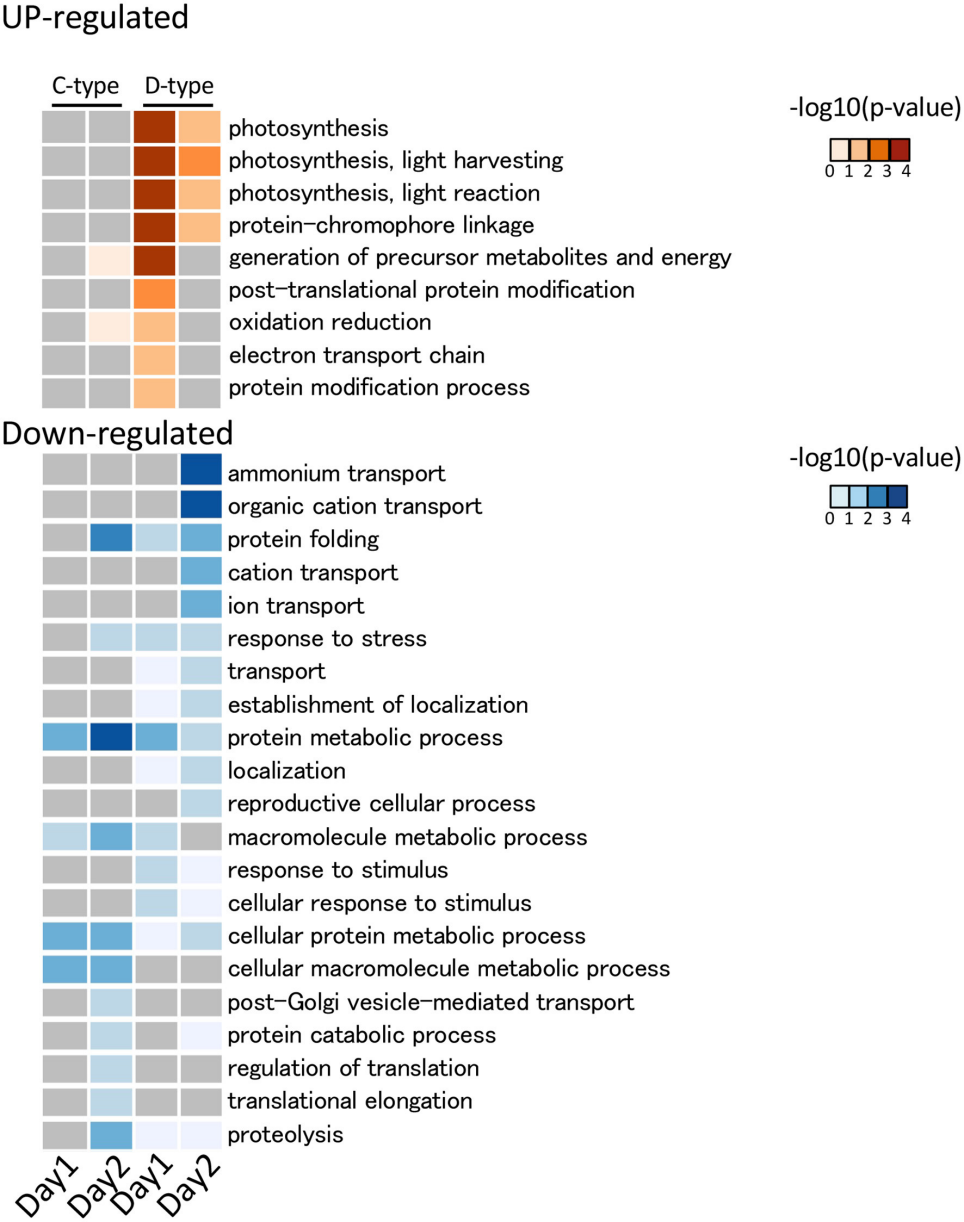
GO functional analysis of Symbiodiniaceae DEGs detected using DAVID. The detected significantly (*p* < 0.05) enriched GO terms in biological process are shown. The legend shows the color scaling with negative log10 *p*-value. In the C-type (*Cladocopium*), no significantly enriched GO term was detected in the upregulated DEG, while in D-type (*Durusdinium*), some photosynthesis-related GO terms were detected as upregulated DEGs. Among the downregulated DEGs, a GO term related to protein folding was enriched in both Symbiodiniaceae.

**Figure 6:**
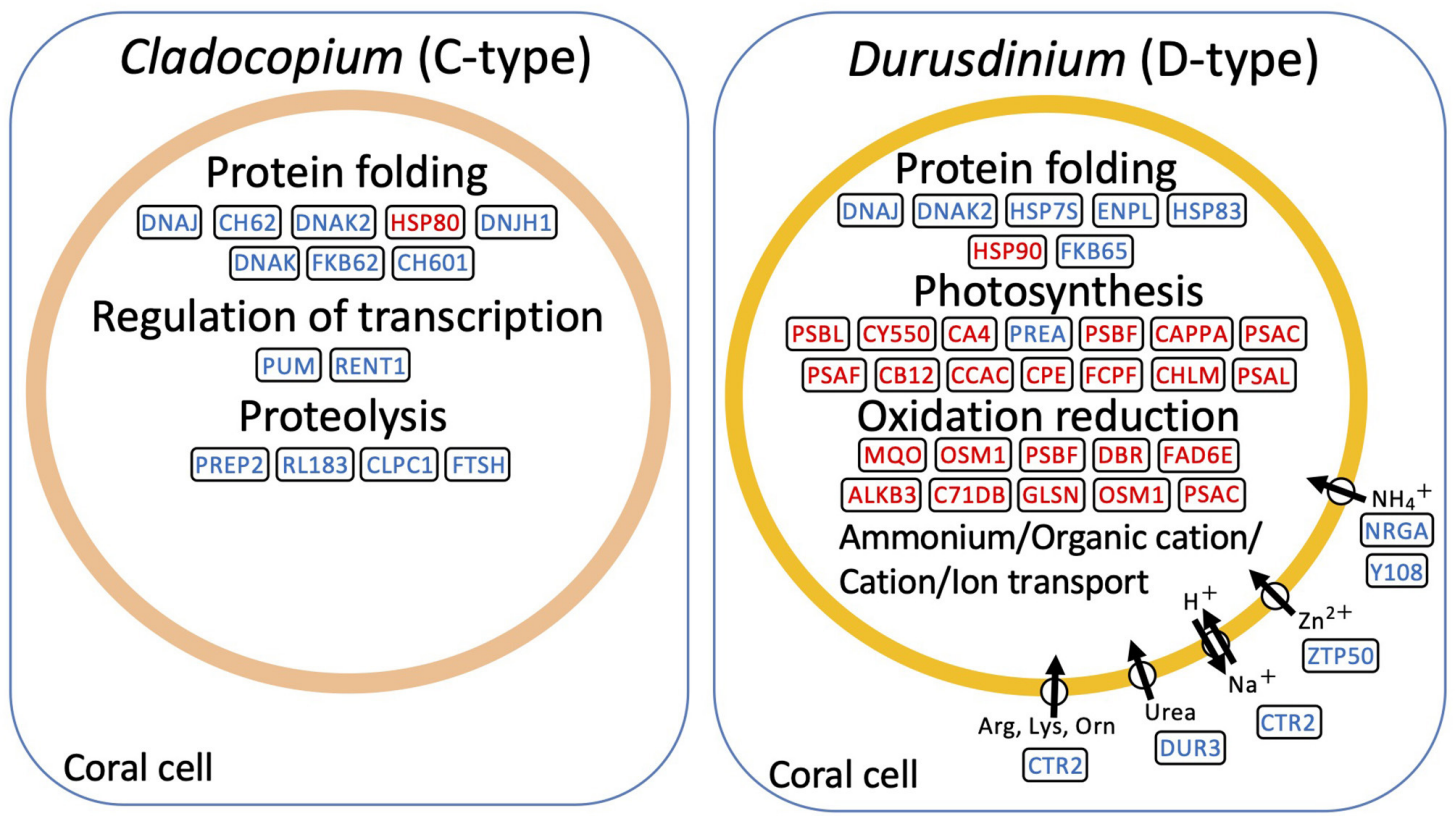
Schematic presentation of representative genes involved in high temperature and strong light stress based on RNA-seq data of symbiont, Cladpcopium (C-type) and Durusdinium (D-type). The representative enriched GO and their related genes were shown. Upregulated transcripts during bleaching are shown in red; downregulated transcripts are indicated in blue.

*De novo* assembly of C- and D-type symbionts was performed, respectively, using FASTQ data of C-and D-corals, which resulted in the identification of 44,446 contigs (DDBJ/ENA/GenBank accession codes ICPI01000001–ICPI01003338) from C-type and 20,368 contigs (DDBJ/ENA/GenBank accession codes ICPJ01000001–ICPJ01004504) from D-type (Supplementary Tables S1, S2). FASTQ reads of C- and D-corals were aligned to candidate each symbiont (C-type and D-type) derived contigs, respectively. Then, the expression levels of each contig were estimated from FPKM values. The expression pattern of each contig was then compared between the control and stress conditions (day 1 or 2) using edgeR and the TCC program (false discovery rate <0.05). In total, 117 and 56 algal DEGs were detected in C- and D-type in stress day 1, and in stress day 2, 109 DEGs and 296 DEGs were detected (Supplementary Figure S6). Of them, 204 C-type and 321 D-type DEGs had protein functional annotations (BLASTX e-value <10e-4). GO enrichment analysis was performed based on the DEG annotations detected for each treatment and algae type. The 29 enriched GO terms (*p*-value <0.05, Fisher's exact test) are shown in Figure 5. Figure 6 shows typical GO terms of C-type and D-type symbionts and their related genes (since the same contigs were shared by GO terms with similar names, representative GOs with high originality were summarized in Figure 6). The stress response of the C-type was characterized by GO terms related to “regulation of translation” and “proteolysis.” For the D-type, GO terms associated with “photosynthesis” were upregulated, while those associated with “ion transport” and “protein folding” were downregulated. The DEGs indicated that several types of transcripts coding chaperon proteins were downregulated in both C-type and D-type. In addition, D-type had many DEGs related to photosynthesis that were upregulated under stress conditions.

## Discussion

In this study, corals associated with C-type or D-type symbionts were incubated under coral bleaching conditions (increasing temperature and strong light conditions), and their stress response patterns and transcriptomic changes were investigated. By the stress treatment, a decrease in symbiotic cell density, which is a typical coral bleaching phenomenon, was observed. Photosynthetic activity (*F*_*v*_*/F*_*m*_) of both Symbiodiniaceae immediately decreased, and the D-type maintaining a slightly higher *F*_*v*_*/F*_*m*_ than the C-type under stress conditions. This is similar to the reports that the degree of *F*_*v*_*/F*_*m*_ decline in the D-type was smaller than in the C-type under strong light conditions (Yuyama et al., 2016). These results were suggested that D-corals have slightly more stress-resistant properties than C-corals. To examine the difference in stress response between C- and D-corals, we performed transcriptome analyses and investigated DEGs between stress treatments and non-stressed control. As a result, we identified 9,884 annotated DEGs derived from corals and 204 and 321 annotated DEGs derived from Cladocopium and Durusdinium. First, we focus on coral genes and discuss the major pathways commonly detected in C- and D-type coral (Figure 3), and then, we focused on the unique gene expression changes of each coral (Figure 4). Our RNA-seq results were limited by insufficient replication (two replicates, each containing ~15 corals). For that reason, rather than focusing on the response of a single gene, we mainly focused on the phenomena detected by GO enrichment analysis of the DEGs. Algal DEGs detected from RNA-seq analyses were lower than the number of host corals, but typical stress-responsive genes, such as genes coding molecular chaperon proteins, were detected. The final section of the discussion focused on the response of symbiotic algae. Transcriptome data on day 2 were mainly used for discussion, because the data on day 1 are in the process of temperature increasing and a lesser number of DEGs were detected.

### Common Transcriptomic Reactions of Corals Between C- and D-Type Corals

#### ER Stress, Apoptosis, and Mitophagy

A total of 107 DEGs involved in “Protein processing in endoplasmic reticulum” were detected in the corals exposed to stresses. These genes have been reported in the previous studies and are considered to be a major molecular response associated with the bleaching response (Oakley et al., 2017; Ruiz-Jones and Palumbi, 2017). These enormous changes in gene expression associated with the unfolded protein response (ER-stress) indicate that corals suffer from increased protein denaturation during the bleaching process. In addition, other processes related to ER stress, such as mitophagy and apoptosis-like responses, were also detected by GO enrichment and KEGG pathway analyses. Mitophagy is a response in which damaged mitochondria are degraded by autophagy and a similar response has been observed in *Exaiptasia* (a model animal of coral–algal endosymbiosis) under high temperature conditions (Dunn et al., 2012). The caspase-mediated apoptosis has also been reported to be involved in bleaching phenomena (Tchernov et al., 2011). This series of responses, such as ER stress, mitophagy, and apoptosis, is likely caused by ROS generated from the photoinhibition of endosymbiotic alga under high temperature and strong light conditions (Lesser, 2011). These ROS attack protein in corals, and this might increase the expression of genes involved in ER stress, mitophagy, and apoptosis, which leads to the coral bleaching.

#### Endocytosis

Expression of genes related to ER stress or mitophagy could also link to the upregulation of Rab 7 protein, which is responsible for the fusion of lysosomes and endosomes (Figure 3). In *Aiptasia*, a Rab 7 homolog is located in putative late endocytic and phagocytic compartments containing either heat-killed or photosynthesis-impaired symbionts (Chen et al., 2003). The Rab 7 homolog may be involved in the digestion of denatured alga; our results that show the upregulation of Rab 7 might be a sign of the bleaching process. In addition to Rab 7, various endocytosis-related DEGs were identified. The expression of Rab proteins, charged multivesicular body proteins (CHMPs), and vacuolar protein sorting (VPS) proteins, which are involved in endocytic trafficking and endosomal sorting, increased under the stress conditions. Less is known about the functions of endocytosis-related genes involved in coral bleaching except for the fact that Rab protein is localized in the symbiosome membrane (Chen et al., 2003, 2005; Song et al., 2015). In plant–microbe symbioses, however, the regulation of the expression of membrane trafficking proteins, such as VPS, causes shrinkage of vacuoles and, in turn, is involved in maturation of the symbiosome membrane (Gavrin et al., 2014). In the case of corals, these fluctuations in endocytosis-related genes such as VPS, Rab, and CHMP will be also important for understanding the status of symbiosome membranes during coral bleaching.

#### Metabolic Processes

Stress exposure reduces the expression of genes involved in the generation of acetyl-CoA from glycolysis (Figure 3). It also reduces the expression of the fatty acid biosynthetic pathway that consumes acetyl-CoA; by contrast, it increases the expression of L-lysine and fatty acid metabolism pathways that generate acetyl-CoA. In other words, the expression levels of L-lysine and fatty acid metabolism-related genes increase to enhance the production of acetyl-CoA under stressed conditions in which less acetyl-CoA is generated through glycolysis. These findings suggest that during bleaching, corals maintain their energy-producing pathway, in which acetyl-CoA enters the TCA cycle, by supplementing acetyl-CoA production using metabolic pathways other than glycolysis. In addition to those mentioned above, other stress response processes, which include cytochrome c release, the heme biosynthetic pathway, arginine and proline metabolism, valine isoleucine, and the chitin metabolic pathway degradation, were also detected. Our results suggest that these metabolic processes are susceptible to coral bleaching or symbiotic state with algae.

#### DNA Replication

The expression of genes involved in DNA replication decreased under stress conditions; that is, genes encoding enzymes such as DNA polymerase, helicase, RNase, and ligase were drastically decreased. The downregulation of DNA replication-related genes can be associated with the “DNA-damage response” induced by heat stress or oxidative stress reported as shown in the study of Kantidze et al. (2016). Corals reportedly suffer DNA damage under oxidative stress caused by photoinhibition of symbionts at high temperatures (Nesa and Hidaka, 2009; Nesa et al., 2012). Our results indicate that, during coral bleaching, DNA damage becomes more serious due to the reduced ability to repair DNA (Supplementary Figure S7).

#### Signaling Pathways

The calcium signaling pathway is a typical gene set known to change in corals at high temperatures (Desalvo et al., 2008; Rosic et al., 2015; Weston et al., 2015). Our RNA-seq data showed decreased expression of genes involved in the calcium signaling pathway, which indicated that under high temperature and strong light conditions, the expression levels of molecules that sense calcium were deceased, and expression levels of calmodulin kinase, which involved in modification of cell functions, also decreased. In addition, signaling systems that control cell proliferation that includes PI3K-AKT, insulin signaling, and mTOR pathways were upregulated under the stress conditions. The PI3K-AKT and mTOR pathways are known to relieve the stress induced by ROS (Yu and Cui, 2016). The upregulation of these gene sets in bleached corals may be attributable to the corals' response to damage caused by ROS stress. The expression of similar pathways has been reported to increase in corals under the influence of light pollution (Rosenberg et al., 2019), and therefore, the upregulation of these pathways could be an indicator of coral stress.

### Unique Gene Expression Changes in C-Corals and D-Corals

#### Vitamin B Biosynthesis

Various genes involved in vitamin B metabolism were specifically downregulated in each of the two corals; DEGs related to riboflavin metabolism were detected in C-corals, while DEGs related to folate and thiamine metabolism were detected in D-corals. Vitamin B is an essential supplement for growing alga (Croft et al., 2005; Agostini et al., 2009; Helliwell et al., 2016), which includes Symbiodiniaceae. These unique DEGs involved in vitamin B metabolism, whose expression levels were decreased with decreasing symbiont density on day 2 (although changes in symbiont density are not statistically significant), imply that each symbiont requires different B vitamins. The present results support the connection between symbiont alga and host vitamin B metabolism and revealed symbiote specificity for various B vitamins.

#### TRP Channels

The expression change of TRP channels is noteworthy because the channels have been extensively characterized as thermosensors (Schepers and Ringkamp, 2009; Samanta et al., 2018). The coral TRP channel, homologous to TRPA1, TRPV5/6, and TRPM3, increased in stress-tolerant D-corals under high temperature and strong light conditions, while C-corals showed few changes regarding these proteins. In general, these channels are known to control the entry of calcium that causes an internal signal to regulate cell activities (Schepers and Ringkamp, 2009; Samanta et al., 2018). The symbiont-associated altered expression levels of TRP and their function in corals are the interesting issues to be addressed in the future and may help us to understand the connection between symbiont type and temperature resistance of the host.

### Transcriptomic Changes of Symbiont

The different stress responsiveness between C- and D-type Symbiodiniaceae in corals was also detected by RNA-seq analysis. The photosynthesis-related GO term was enriched in upregulated DEGs in the D-type (Figure 5). By contrast, in the C-type, only a few photosynthesis-related were differentially expressed (Figure 6). The altered expression of transcripts related to the photosynthetic reaction center suggested that algae are affected by photoinhibition, which damages the photosynthetic system under strong light conditions (Constant et al., 1997). In addition, several genes involved in ion transport were downregulated in the D-type (Figures 5, 6). These gene expression changes are likely to reflect an increase or decrease in the amount of substances transported from corals, and downregulation of ammonia transport proteins may reflect a decrease in the transport of ammonia received by symbiotic algae from the host (Pernice et al., 2012). Other transporter that includes zinc transporter and cationic amino acid transporter is also downregulated in D-type, which suggested that D-type cells usually utilize these substances, but their utilization is reduced under stress conditions.

The RNA-seq analysis that focusses on the *in hospite* Symbiodiniaceae detected protein-folding-related transcripts as DEGs in both the C- and D-types, most of which were downregulated by stress exposure (Figure 6). The expression pattern of these transcripts was previously reported in the symbiont of *Acropora millepora* and is considered to be a general symbiont response (Gierz et al., 2017). Interestingly, our results showed that a few protein-folding-related transcripts were upregulated. The upregulation of these heat shock proteins might be associated with increased stress tolerance of Symbiodiniaceae (Mayer and Bukau, 2005; ul Haq et al., 2019). In the bleaching situation, these upregulated chaperone transcripts are thought to be involved in maintaining the functional stability of C- and D-type symbionts.

In summary, the differential expression of many bleaching stress-related genes in corals was detected, and representative changes included upregulation of genes associated with ER stress, apoptosis, mitophagy, endocytosis, PI3K/AKT/mTOR signaling, and metabolic processes related to acetyl-CoA, as well as decreased DNA replication. In addition, the use of corals colonized with two different types of symbionts revealed differences in the expression patterns of TRP channel genes and vitamin B metabolic genes, which shows the influence of symbiont algal type on the coral host. In the algal DEGs, typical stress response genes, such as gene related to molecular chaperon proteins and gene related to photosystem, were detected. Some of those DEGs were frequently detected in the D-type, which has relatively stress-resistant properties. To reveal how the above-mentioned genes are involved in coral bleaching, further examination of the localization of the proteins encoded by each gene and the confirmation of their functions by gene knockdown will be needed. We have summarized typical pathways, but the published transcriptomic data include more information than discussed here. It is expected that these data will help to reveal the underlying mechanisms of coral bleaching in the future studies.

## Data Availability Statement

The raw fastq files for the RNA-seq libraries were deposited in the DDBJ Sequence Read Archive (DRA) under the accession number DRA008078 under DDBJ bioproject PRJDB6866. All nucleotide sequences assembled in this study have been deposited in the DDBJ/ENA/GenBank: A. solitaryensis assembly (accession codes ICPH01000001-ICPH01040036), Cladocopium assembly (accession codes ICPI01000001–ICPI01003338), and Durusdinium assembly (accession codes ICPJ01000001–ICPJ01004504; https://www.ddbj.nig.ac.jp/news/en/190927_2-e.html).

## Author Contributions

IY: study conception, design, data collection, and draft manuscript preparation. TH: statistical analysis, drafting, and writing. HT and TM: sample preparation. KI: drafting and writing. All authors contributed to the article and approved the submitted version.

## Funding

This work was supported by research grants from the Japan Society for the Promotion of Science (IY, No. 14J40135 and No. 19H03026) and the Environment Research and Technology Development Fund (No. 4–1806) from the Ministry of the Environment, Japan.

## Conflict of Interest

The authors declare that the research was conducted in the absence of any commercial or financial relationships that could be construed as a potential conflict of interest.

## Publisher's Note

All claims expressed in this article are solely those of the authors and do not necessarily represent those of their affiliated organizations, or those of the publisher, the editors and the reviewers. Any product that may be evaluated in this article, or claim that may be made by its manufacturer, is not guaranteed or endorsed by the publisher.

## Supplementary Material

**Supplementary Material:** Lists of all contigs identified as *Acropora solitaryensis* (Data sheet 1.csv) -, *Cladocopium* (Data sheet 2.csv) -and *Durusdinium* (Data sheet 3.csv) -derived contigs. Annotation results of all contigs in the Swiss-Prot and non-redundant protein databases; normalized transcript expression levels of each contig are also shown.
