# Supplementary material for: Metatranscriptomic Analysis of Corals Inoculated With Tolerant and Non-Tolerant Symbiont Exposed to High Temperature and Light Stress
Source: Front Physiol. 2022 Apr 11;13:806171. doi: 10.3389/fphys.2022.806171 (PMC9037784; doi:10.3389/fphys.2022.806171)
Supplement: Supplementary file 4 [file Data_Sheet_4.PDF]

Supplementary Information

Metatranscriptomic Analysis of corals inoculated with tolerant and non-tolerant symbiont exposed to high temperature and light stress

Ikuko Yuyama, Tomihiko Higuchi, Takuma Mezaki, Hisako Tashiro, Kazuho Ikeo

Tale of Content

Supplementary Figures

Fig. S1. Schematic diagram of the incubation experiments.······························3

Fig. S2. Numbers of corals used for each analysis.······································4

Fig. S3. Restriction fragment length polymorphism (RFLP) (28S) band patterns of Symbiodiniaceae in juvenile corals inoculated with *Cladocopium* and *Durusdinium*. ···5

Fig. S4. Symbiont density in corals associated with *Cladocopium* (C-corals) and those associated with *Durusdinium* (D-corals) and photographs of C-corals and D-corals exposed to heat and light stress.···6

Fig. S5.MA plot for edgeR for differentially expression analysis on *Acropora solitaryensis associated with Cladocopium or Durusdinium.* ······································7

Fig. S6. MA plot for edgeR for differentially expression analysis on Cladocopium and Durusdinium. ···8

Fig. S7. Expression pattern of genes related to the pathway described in Figure 5.········9~15

Fig. S8. Expression pattern of DEG (differentially expressed genes) related to GO term in figure 6. ···16

Supplementary Table

Table S1. The number of aligned sequences to the database. 17

Table S2. Statistics of obtained Symbiodiniaceae (Cladocopium and Durusdinium) derived contigs and *Acropora solitaryensis* derived contigs. 17

Fig. S1. Schematic diagram of the incubation experiments. (A) Corals were incubated approximately two months after inoculation with *Cladocopium* or *Durusdinium*. To examine the bleaching response, some corals were exposed to stress for 1 or 2 days. (B) In the stress treatment groups, the temperature was gradually increased to 31 °C. For the control populations, corals were maintained at 27°C for approximately 2 months and thenb fixed at the same time as the stress day 2 corals.

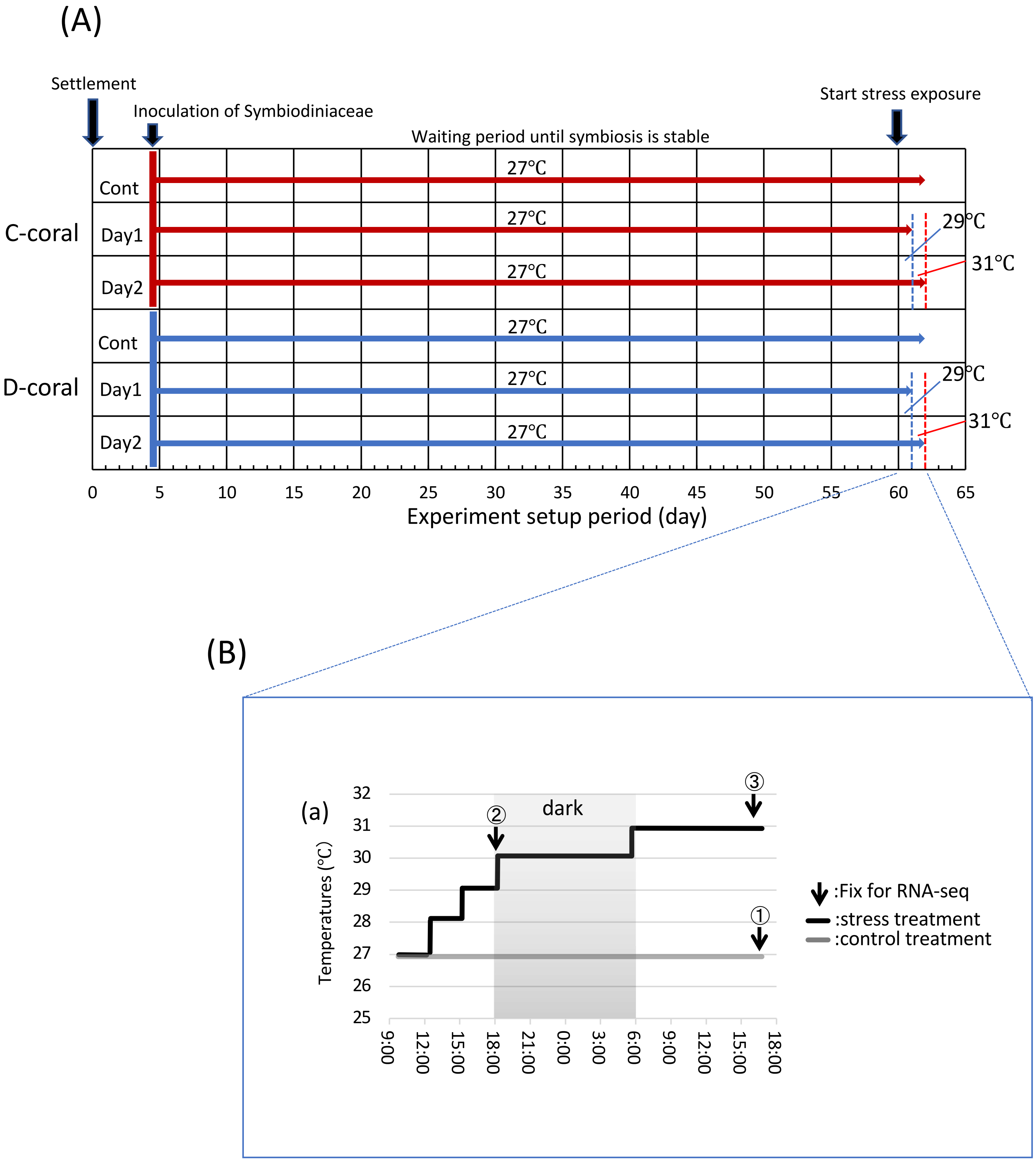

Fig. S2. Numbers of corals used for each analysis. Approximately 200 corals (100 *Cladcopium*-associated and 100 *Durusdinium*-associated corals) were used. These corals were used in stress exposure experiments to investigate symbiot densities and Fv/Fm, and for RNA-Seq analysis, respectively.

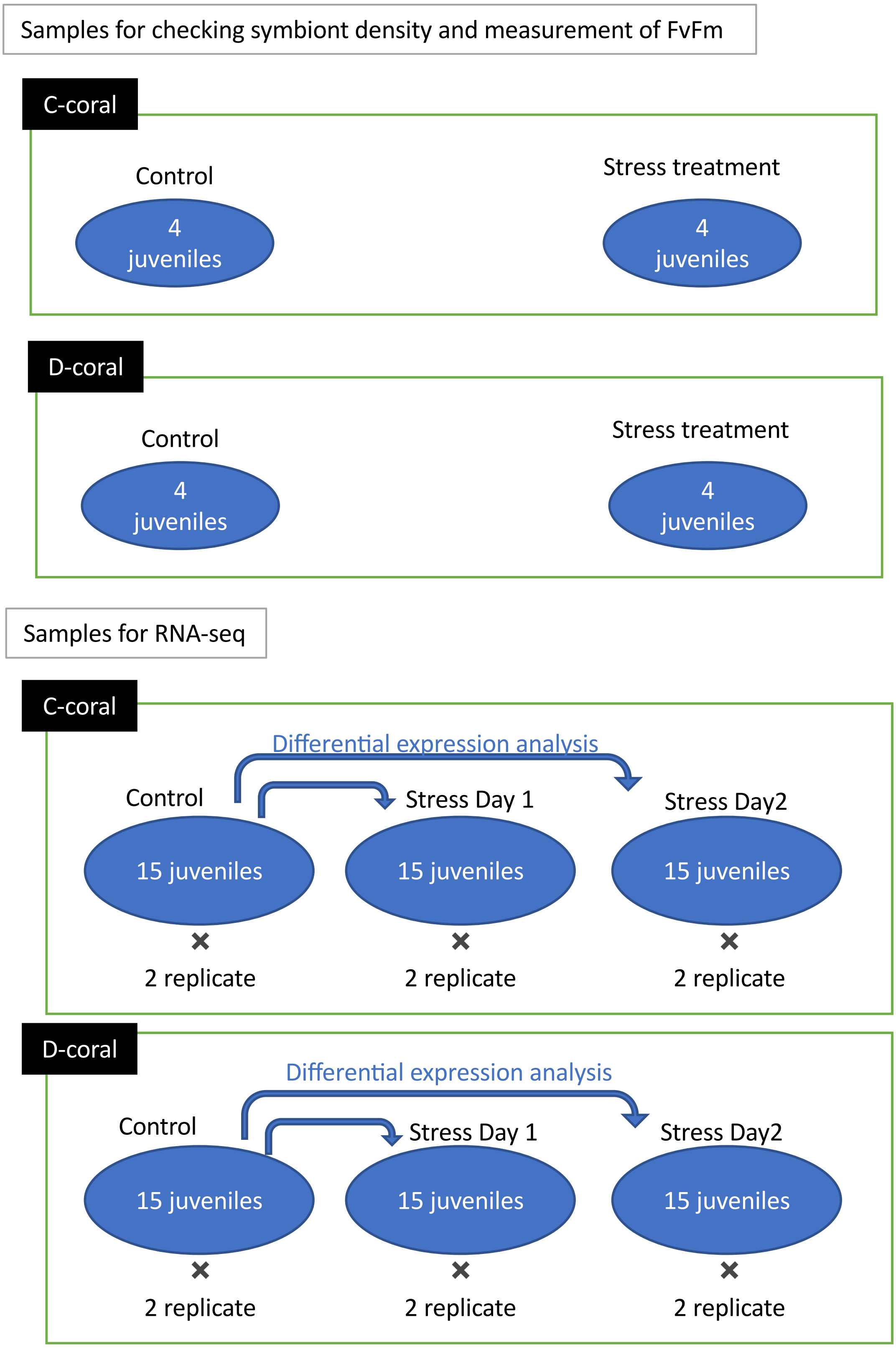

Differential expression analysis were conducted between Stress Day 2 sample and control,, and between stress Day 1 sample and control.

Fig.S3. Restriction fragment length polymorphism (RFLP) (28S) band patterns of Symbiodiniaceae in juvenile corals inoculated with *Cladocopium* and *Durusdinium*. RFLP analysis was performed to verify the genotype of Symbiodiniaceae colonizing corlas before the start of the stress exposure experiments. In RFLP analysis, *Cladocopium* is asslciated with two bands and *Durusdinium* with one band (Yuyama et al., 2014). Electrophoresis bands specific to *Cladocopium* and *Durusdinium* were detected in *A. solitaryensis* inoculated with either Symbiodiniaceae.

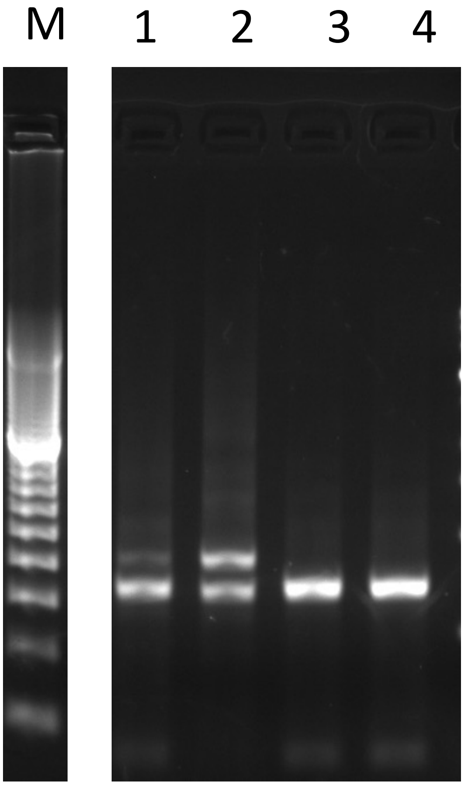

M: 100bp DNA ladder
1 : *Acropora tenuis* juveniles inoculated with clade C
2 : *Acropora solitaryensis* juveniles inoculated with clade C
3 : *Acropora tenuis* juveniles with clade D
4 : *Acropora solitaryensis* juveniles inoculated with clade D

**A. tenuis* is not used in this study.
.

Fig. S4. Symbiont density in corals associated with *Cladocopium* (C-corals) and those associated with *Durusdinium* (D-corals) and photographs of C-corals and D-corals exposed to heat and light stress. The density of the symbiotic algae (*Durusdinium* and *Cladocopium*) in the corals for the control populations and for the 2-day stress treatment are shown. Cell density was calculated based on the surface area of the photographed coral. As the temperature increased, the symbiont number inside the coral body decreased, causing the coral to appear whiter. Scale bars = 0.5mm.

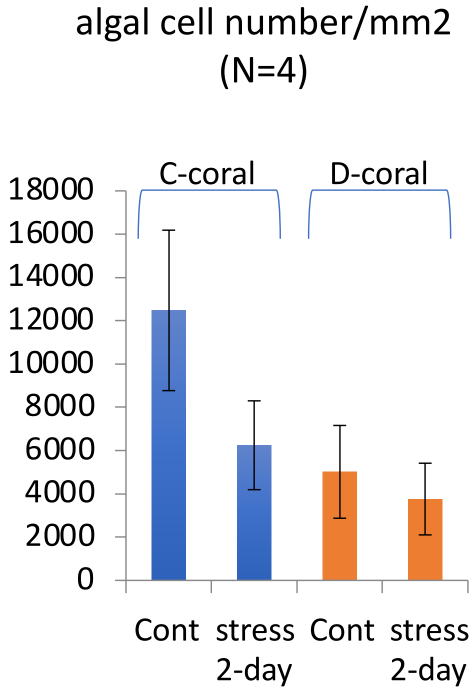

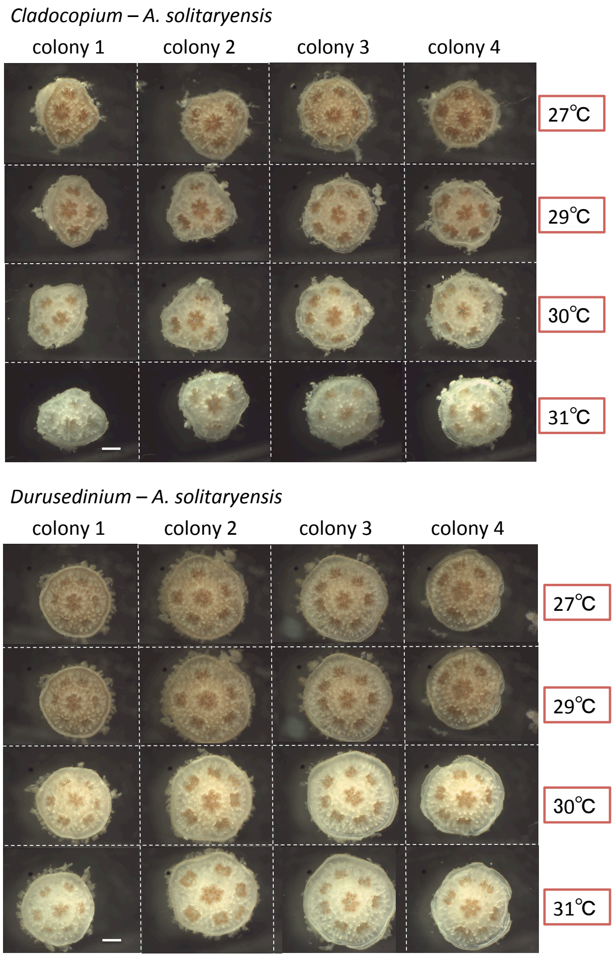

Figure S5, MA plot for edgeR for differentially expression analysis on *Acropora solitaryensis associated with Cladocopium or Durusdinium*. The log2 fold change in stress conditions compared with the control conditions is plotted on the y-axis and the average log2 counts across samples are shown on the x-axis. Isoforms that are significantly different among two conditions less than 0.05 are colored in red, others are colored in gray. The number of detected differentially expressed genes are shown in parentheses.

Corals associated with Cladocopium (C-coral)

stress day 1 (1,685)
(up-regulated : 967, down-regulated : 718)

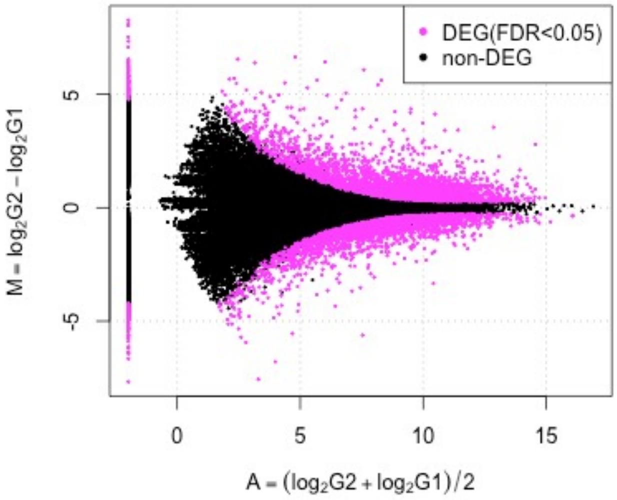

stress day 2 (2,954)
(up-regulated :1,463, down-regulated : 1,491)

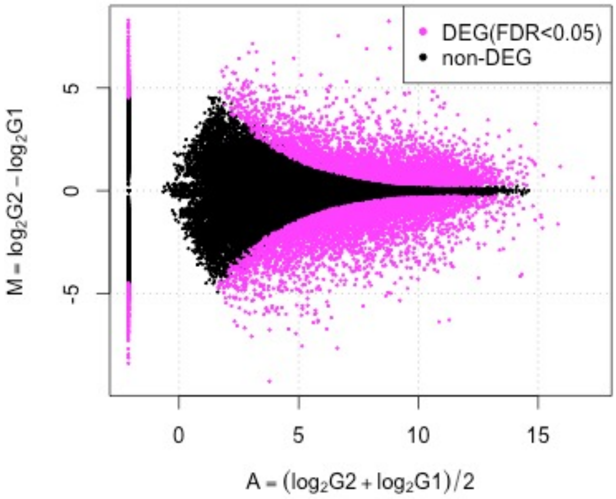

Corals associated with Durusdinium (D-coral)

stress day 1 (1,081)
(up-regulated : 537, down-regulated : 544)

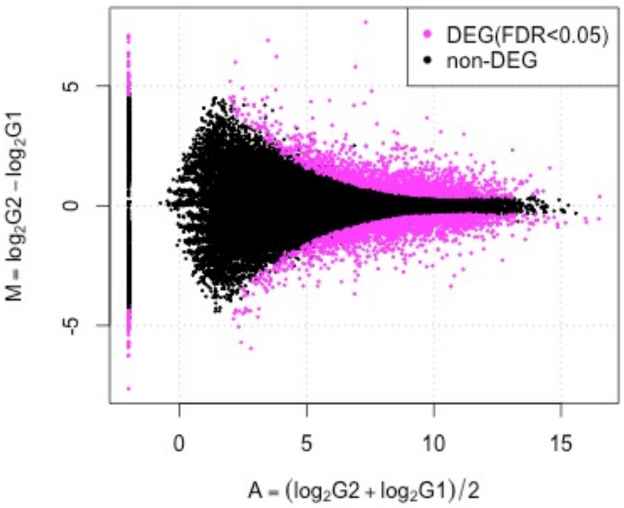

stress day 2 (3516)
(up-regulated : 1,759, down-regulated : 1,757)

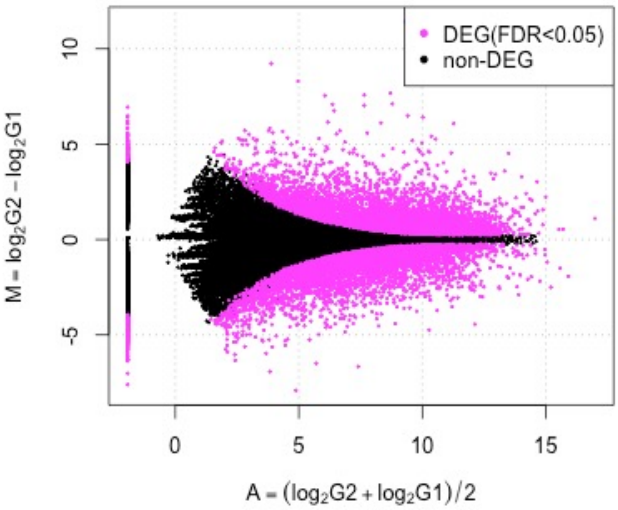

Figure S6, MA plot for edgeR for differentially expression analysis on Cladocopium and Durusdinium. The log2 fold change in stress conditions compared with the control conditions is plotted on the y-axis and the average log2 counts across samples are shown on the x-axis. Isoforms that are significantly different among two conditions less than 0.05 are colored in red, others are colored in gray. The number of detected differentially expressed genes are shown in parentheses.

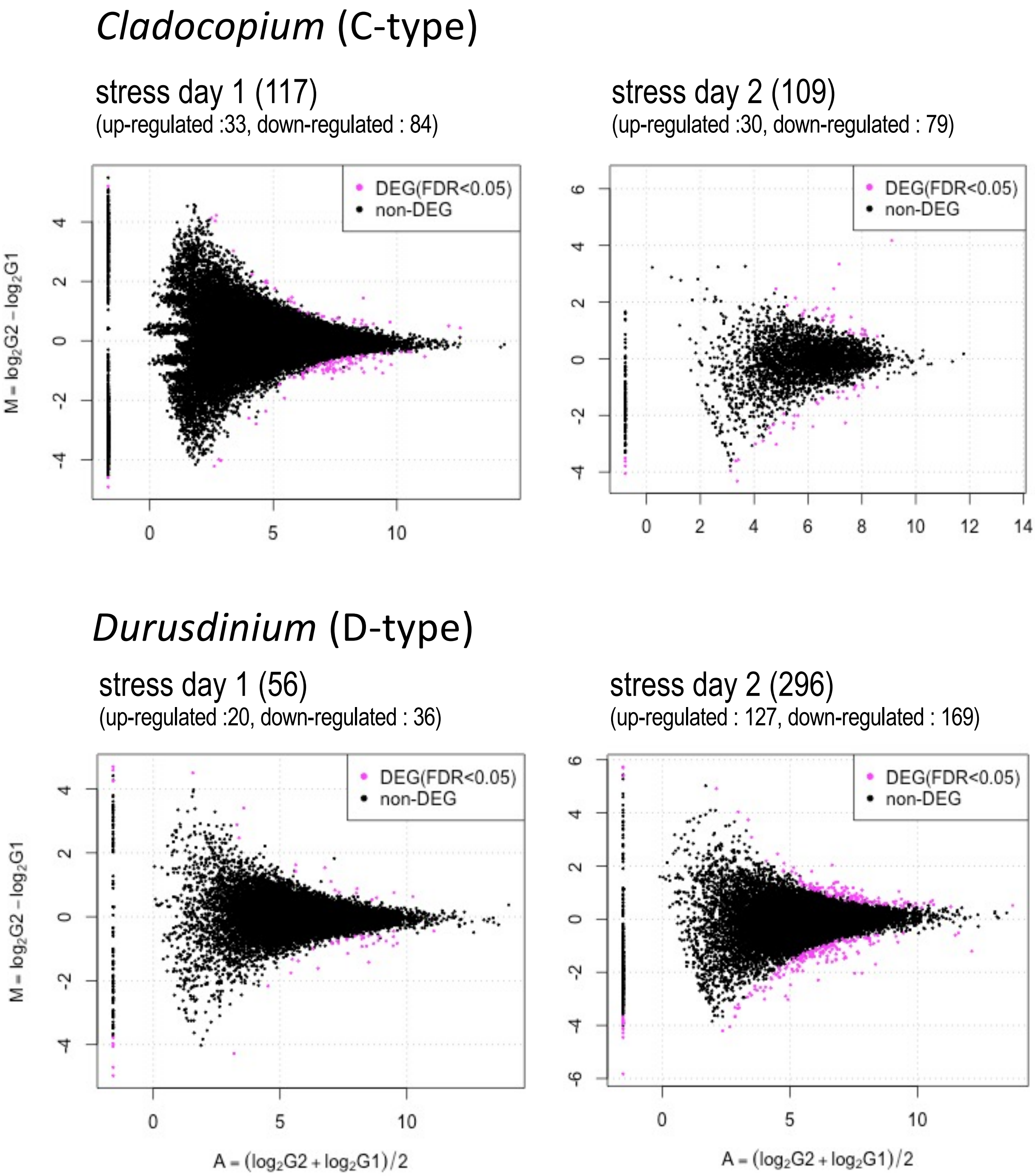

Fig. S7. The heatmap shows the expression level of genes related to each pathway described in Fig. 5.

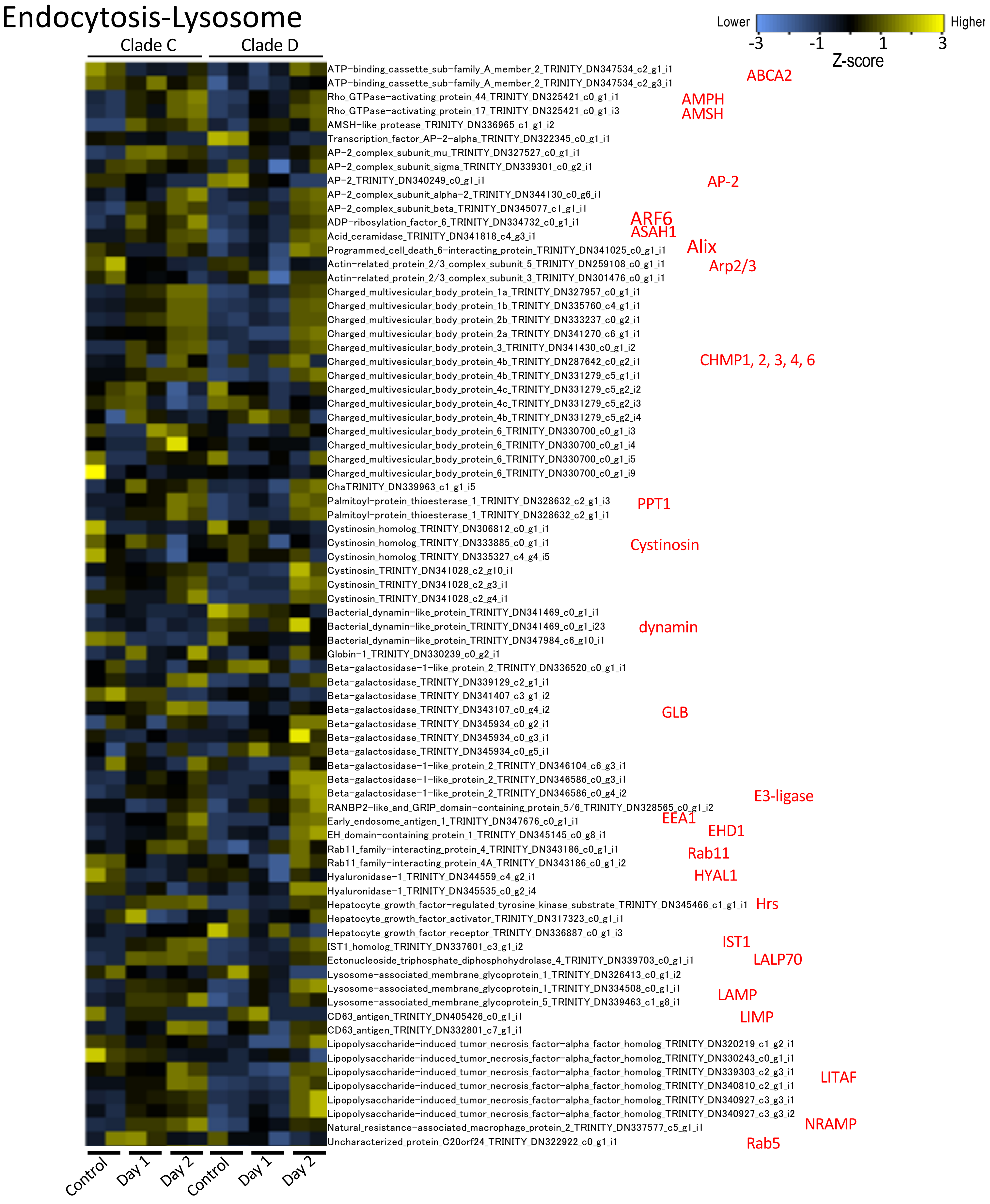

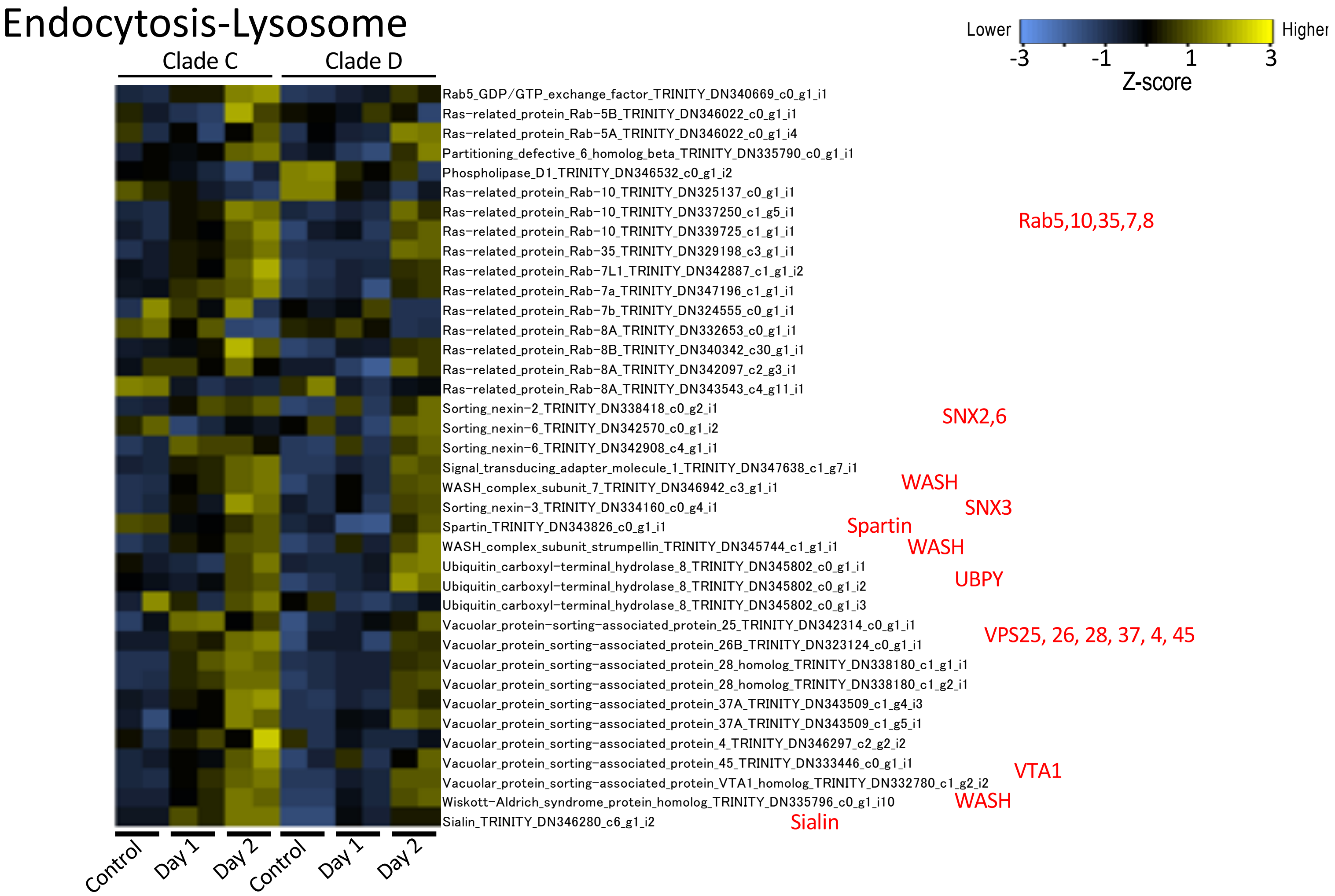
Endocytosis-Lysosome
Clade C
Clade D
Lower
Higher
-3
-1
1
3
Z-score
Rab5_GDP/GTP_exchange_factor_TRINITY_DN340669_c0_g1_i1
Ras−related_protein_Rab−5B_TRINITY_DN346022_c0_g1_i1
Ras−related_protein_Rab−5A_TRINITY_DN346022_c0_g1_i4
Partitioning_defective_6_homolog_beta_TRINITY_DN335790_c0_g1_i1
Phospholipase_D1_TRINITY_DN346532_c0_g1_i2
Ras−related_protein_Rab−10_TRINITY_DN325137_c0_g1_i1
Ras−related_protein_Rab−10_TRINITY_DN337250_c1_g5_i1
Ras−related_protein_Rab−10_TRINITY_DN339725_c1_g1_i1
Ras−related_protein_Rab−35_TRINITY_DN329198_c3_g1_i1
Ras−related_protein_Rab−7L1_TRINITY_DN342887_c1_g1_i2
Ras−related_protein_Rab−7a_TRINITY_DN347196_c1_g1_i1
Ras−related_protein_Rab−7b_TRINITY_DN324555_c0_g1_i1
Ras−related_protein_Rab−8A_TRINITY_DN332653_c0_g1_i1
Ras−related_protein_Rab−8B_TRINITY_DN340342_c30_g1_i1
Ras−related_protein_Rab−8A_TRINITY_DN342097_c2_g3_i1
Ras−related_protein_Rab−8A_TRINITY_DN343543_c4_g11_i1
Sorting_nexin−2_TRINITY_DN338418_c0_g2_i1
Sorting_nexin−6_TRINITY_DN342570_c0_g1_i2
Sorting_nexin−6_TRINITY_DN342908_c4_g1_i1
Signal_transducing_adapter_molecule_1_TRINITY_DN347638_c1_g7_i1
WASH_complex_subunit_7_TRINITY_DN346942_c3_g1_i1
Sorting_nexin−3_TRINITY_DN334160_c0_g4_i1
Spartin_TRINITY_DN343826_c0_g1_i1
WASH_complex_subunit_strumpellin_TRINITY_DN345744_c1_g1_i1
Ubiquitin_carboxyl−terminal_hydrolase_8_TRINITY_DN345802_c0_g1_i1
Ubiquitin_carboxyl−terminal_hydrolase_8_TRINITY_DN345802_c0_g1_i2
Ubiquitin_carboxyl−terminal_hydrolase_8_TRINITY_DN345802_c0_g1_i3
Vacuolar_protein−sorting−associated_protein_25_TRINITY_DN342314_c0_g1_i1
Vacuolar_protein_sorting−associated_protein_26B_TRINITY_DN323124_c0_g1_i1
Vacuolar_protein_sorting−associated_protein_28_homolog_TRINITY_DN338180_c1_g1_i1
Vacuolar_protein_sorting−associated_protein_28_homolog_TRINITY_DN338180_c1_g2_i1
Vacuolar_protein_sorting−associated_protein_37A_TRINITY_DN343509_c1_g4_i3
Vacuolar_protein_sorting−associated_protein_37A_TRINITY_DN343509_c1_g5_i1
Vacuolar_protein_sorting−associated_protein_4_TRINITY_DN346297_c2_g2_i2
Vacuolar_protein_sorting−associated_protein_45_TRINITY_DN333446_c0_g1_i1
Vacuolar_protein_sorting−associated_protein_VTA1_homolog_TRINITY_DN332780_c1_g2_i2
Wiskott−Aldrich_syndrome_protein_homolog_TRINITY_DN335796_c0_g1_i10
Sialin_TRINITY_DN346280_c6_g1_i2
Rab5,10,35,7,8
SNX2,6
WASH
SNX3
Spartin
WASH
UBPY
VPS25, 26, 28, 37, 4, 45
VTA1
WASH
Sialin
Control
Day 1
Day 2
Control
Day 1
Day 2

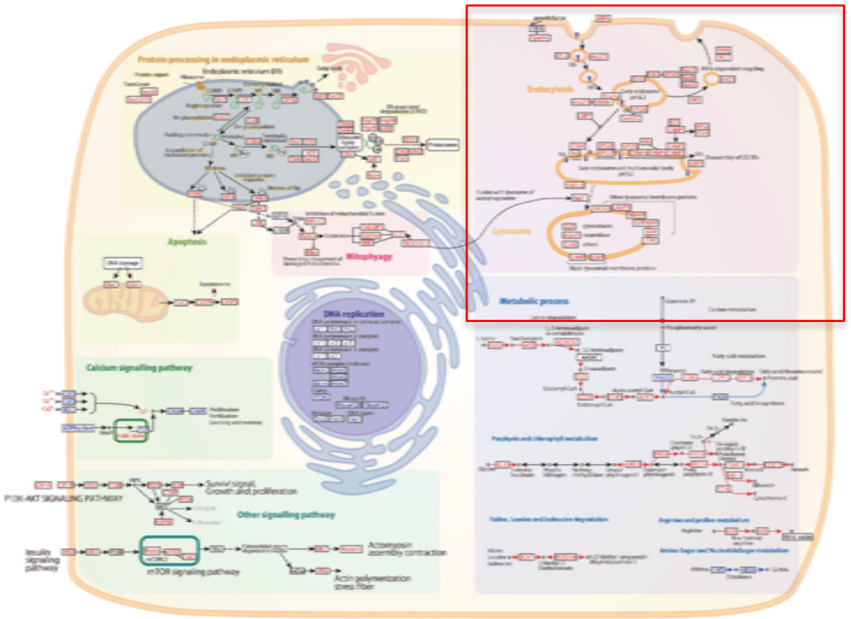

Protein processing in endoplasmic reticulum

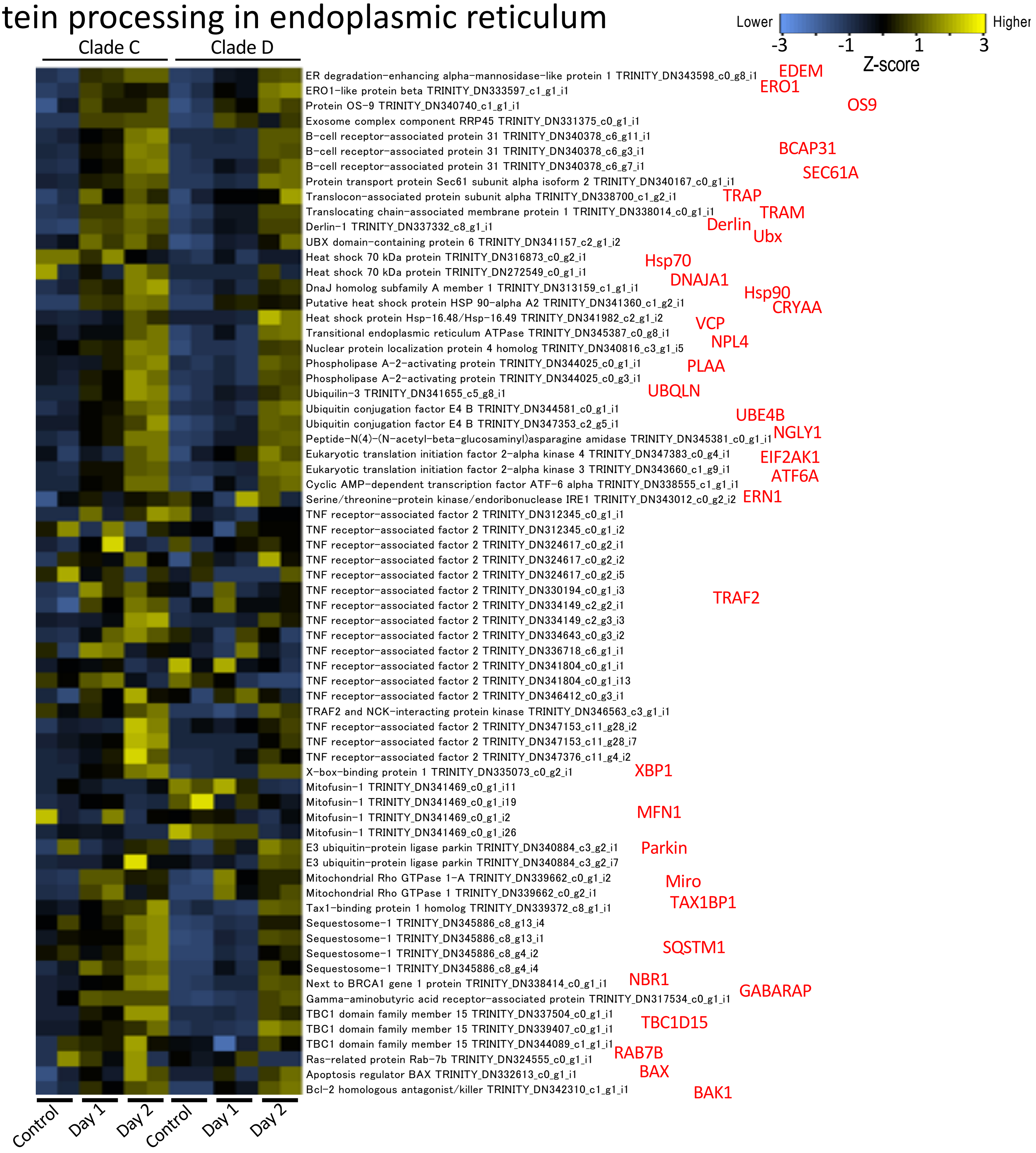

Protein processing in endoplasmic reticulum

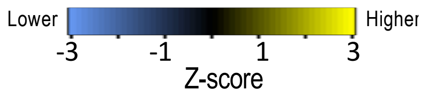

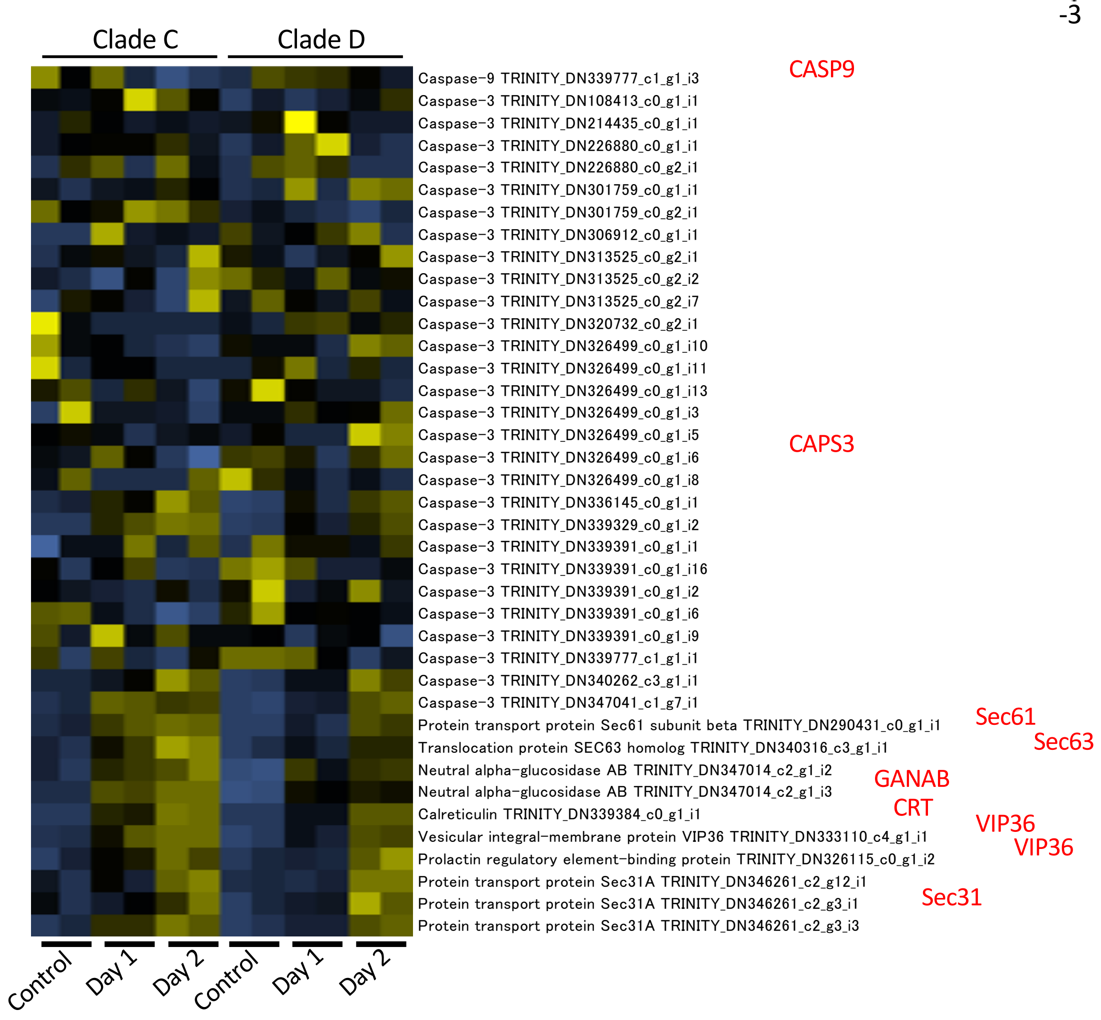

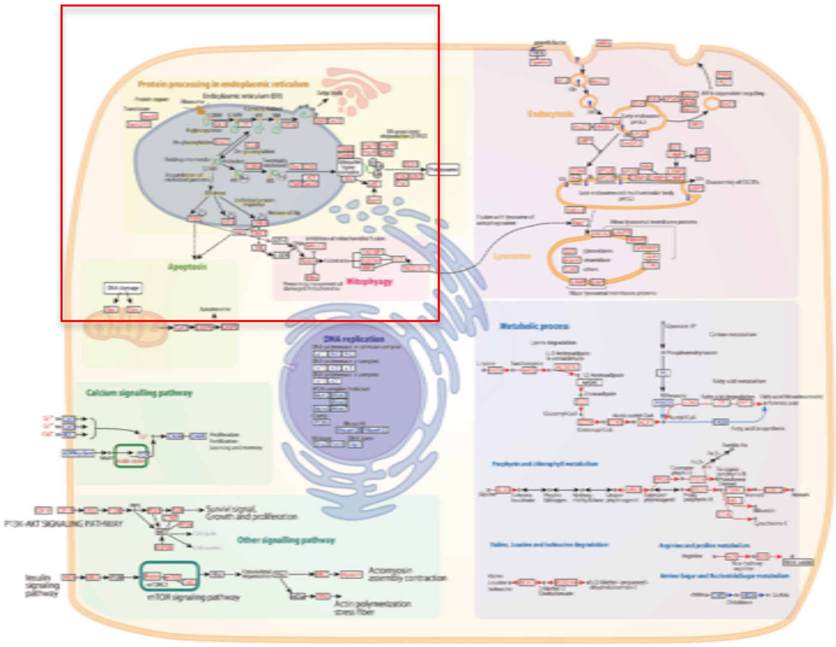

Calcium signaling pathway

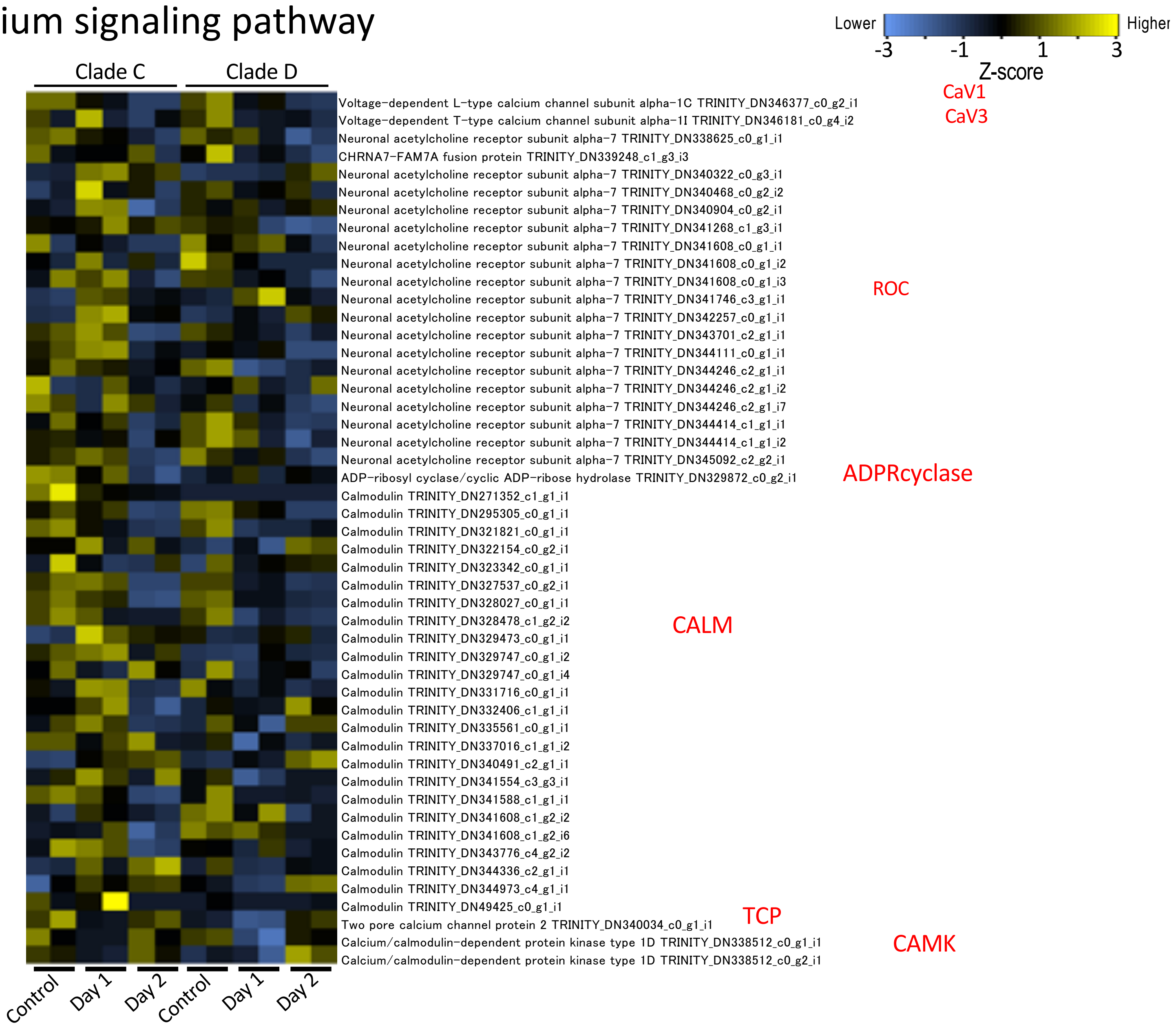

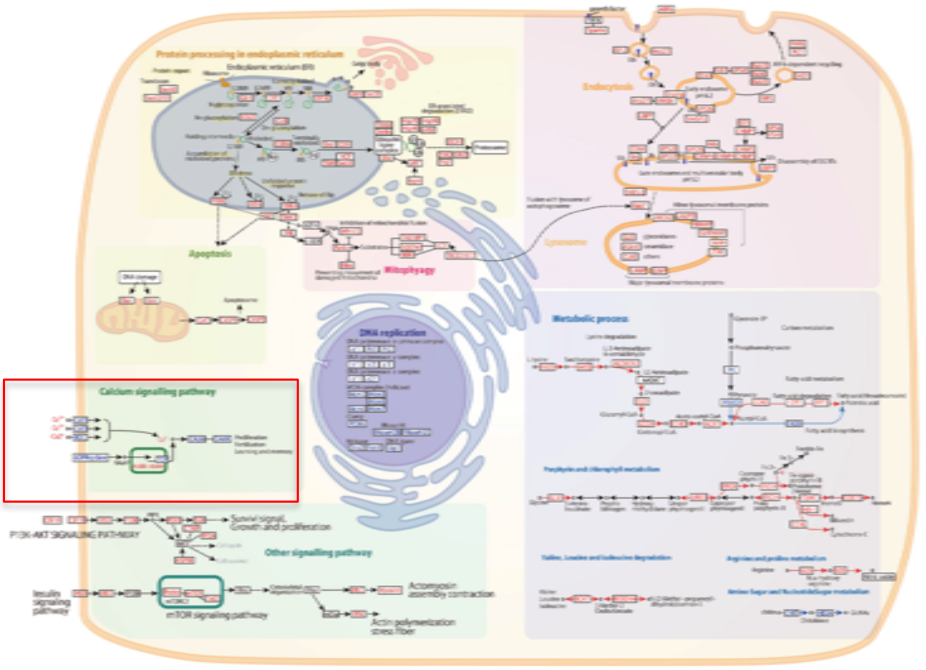

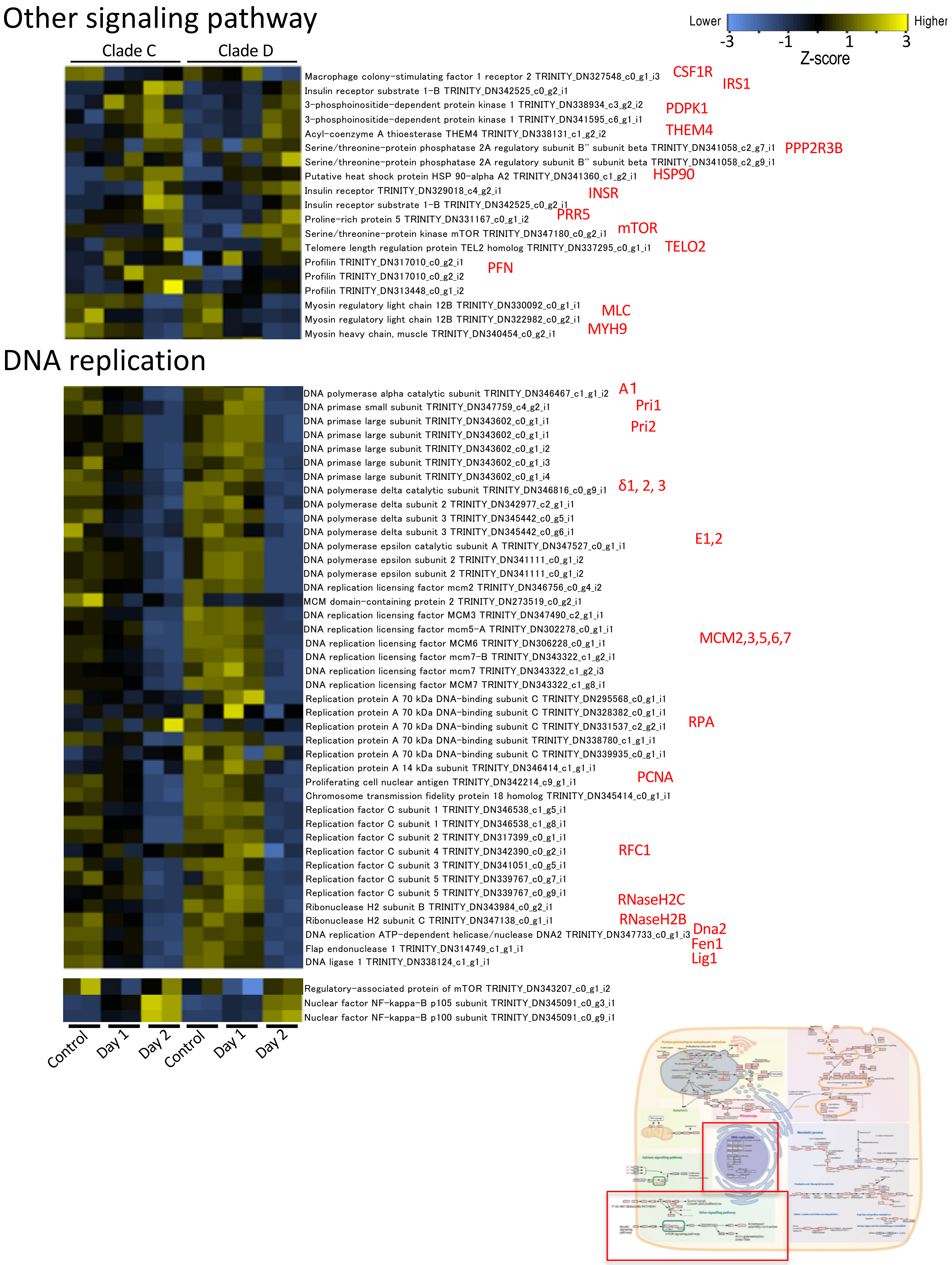
Other signaling pathway
Lower
Higher
-3
-1
1
3
Z-score
Clade C
Clade D
Macrophage colony−stimulating factor 1 receptor 2 TRINITY_DN327548_c0_g1_i3
CSF1R
Insulin receptor substrate 1−B TRINITY_DN342525_c0_g2_i1
IRS1
3−phosphoinositide−dependent protein kinase 1 TRINITY_DN338934_c3_g2_i2
3−phosphoinositide−dependent protein kinase 1 TRINITY_DN341595_c6_g1_i1
PDPK1
Acyl−coenzyme A thioesterase THEM4 TRINITY_DN338131_c1_g2_i2
THEM4
Serine/threonine−protein phosphatase 2A regulatory subunit B'' subunit beta TRINITY_DN341058_c2_g7_i1
Serine/threonine−protein phosphatase 2A regulatory subunit B'' subunit beta TRINITY_DN341058_c2_g9_i1
PPP2R3B
Putative heat shock protein HSP 90−alpha A2 TRINITY_DN341360_c1_g2_i1
HSP90
Insulin receptor TRINITY_DN329018_c4_g2_i1
INSR
Insulin receptor substrate 1−B TRINITY_DN342525_c0_g2_i1
Proline−rich protein 5 TRINITY_DN331167_c0_g1_i2
PRR5
Serine/threonine−protein kinase mTOR TRINITY_DN347180_c0_g2_i1
mTOR
Telomere length regulation protein TEL2 homolog TRINITY_DN337295_c0_g1_i1
TELO2
Profilin TRINITY_DN317010_c0_g2_i1
Profilin TRINITY_DN317010_c0_g2_i2
Profilin TRINITY_DN313448_c0_g1_i2
PFN
Myosin regulatory light chain 12B TRINITY_DN330092_c0_g1_i1
Myosin regulatory light chain 12B TRINITY_DN322982_c0_g2_i1
MLC
Myosin heavy chain, muscle TRINITY_DN340454_c0_g2_i1
MYH9
DNA replication
DNA polymerase alpha catalytic subunit TRINITY_DN346467_c1_g1_i2
A1
DNA primase small subunit TRINITY_DN347759_c4_g2_i1
Pri1
DNA primase large subunit TRINITY_DN343602_c0_g1_i1
DNA primase large subunit TRINITY_DN343602_c0_g1_i1
DNA primase large subunit TRINITY_DN343602_c0_g1_i2
DNA primase large subunit TRINITY_DN343602_c0_g1_i3
DNA primase large subunit TRINITY_DN343602_c0_g1_i4
Pri2
DNA polymerase delta catalytic subunit TRINITY_DN346816_c0_g9_i1
DNA polymerase delta subunit 2 TRINITY_DN342977_c2_g1_i1
DNA polymerase delta subunit 3 TRINITY_DN345442_c0_g5_i1
DNA polymerase delta subunit 3 TRINITY_DN345442_c0_g6_i1
δ1, 2, 3
DNA polymerase epsilon catalytic subunit A TRINITY_DN347527_c0_g1_i1
DNA polymerase epsilon subunit 2 TRINITY_DN341111_c0_g1_i2
DNA polymerase epsilon subunit 2 TRINITY_DN341111_c0_g1_i2
E1,2
DNA replication licensing factor mcm2 TRINITY_DN346756_c0_g4_i2
MCM domain−containing protein 2 TRINITY_DN273519_c0_g2_i1
DNA replication licensing factor MCM3 TRINITY_DN347490_c2_g1_i1
DNA replication licensing factor mcm5−A TRINITY_DN302278_c0_g1_i1
DNA replication licensing factor MCM6 TRINITY_DN306228_c0_g1_i1
DNA replication licensing factor mcm7−B TRINITY_DN343322_c1_g2_i1
DNA replication licensing factor mcm7 TRINITY_DN343322_c1_g2_i3
DNA replication licensing factor MCM7 TRINITY_DN343322_c1_g8_i1
MCM2,3,5,6,7
Replication protein A 70 kDa DNA−binding subunit C TRINITY_DN295568_c0_g1_i1
Replication protein A 70 kDa DNA−binding subunit C TRINITY_DN328382_c0_g1_i1
Replication protein A 70 kDa DNA−binding subunit C TRINITY_DN331537_c2_g2_i1
Replication protein A 70 kDa DNA−binding subunit TRINITY_DN338780_c1_g1_i1
Replication protein A 70 kDa DNA−binding subunit C TRINITY_DN339935_c0_g1_i1
Replication protein A 14 kDa subunit TRINITY_DN346414_c1_g1_i1
RPA
Proliferating cell nuclear antigen TRINITY_DN342214_c9_g1_i1
PCNA
Chromosome transmission fidelity protein 18 homolog TRINITY_DN345414_c0_g1_i1
Replication factor C subunit 1 TRINITY_DN346538_c1_g5_i1
Replication factor C subunit 1 TRINITY_DN346538_c1_g8_i1
Replication factor C subunit 2 TRINITY_DN317399_c0_g1_i1
Replication factor C subunit 4 TRINITY_DN342390_c0_g2_i1
Replication factor C subunit 3 TRINITY_DN341051_c0_g5_i1
Replication factor C subunit 5 TRINITY_DN339767_c0_g7_i1
Replication factor C subunit 5 TRINITY_DN339767_c0_g9_i1
RFC1
Ribonuclease H2 subunit B TRINITY_DN343984_c0_g2_i1
RNaseH2C
Ribonuclease H2 subunit C TRINITY_DN347138_c0_g1_i1
RNaseH2B
DNA replication ATP−dependent helicase/nuclease DNA2 TRINITY_DN347733_c0_g1_i3
Dna2
Flap endonuclease 1 TRINITY_DN314749_c1_g1_i1
Fen1
DNA ligase 1 TRINITY_DN338124_c1_g1_i1
Lig1
Regulatory−associated protein of mTOR TRINITY_DN343207_c0_g1_i2
Nuclear factor NF−kappa−B p105 subunit TRINITY_DN345091_c0_g3_i1
Nuclear factor NF−kappa−B p100 subunit TRINITY_DN345091_c0_g9_i1
Control
Day 1
Day 2
Control
Day 1
Day 2

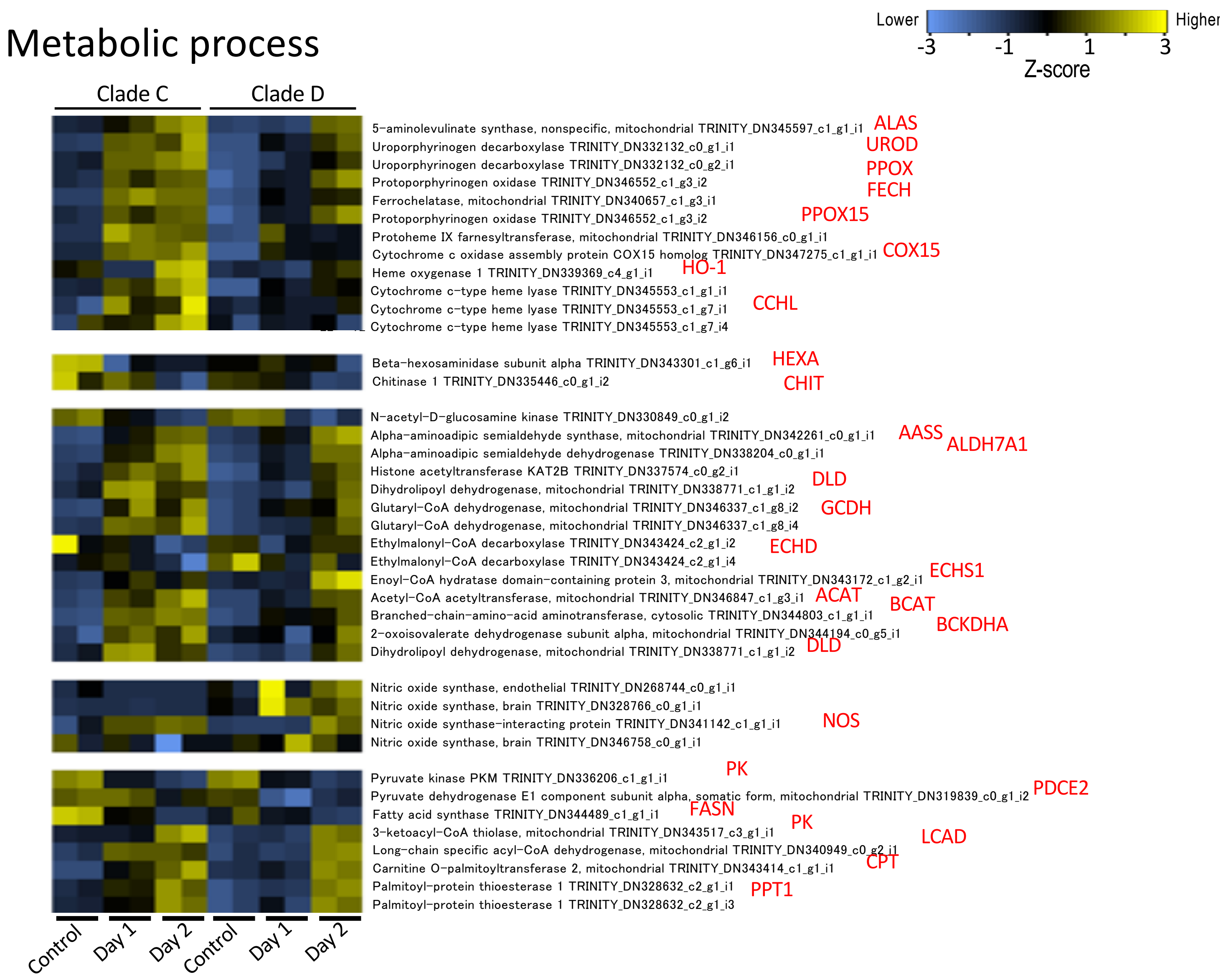
Metabolic process
Lower
Higher
-3
-1
1
3
Z-score
Clade C
Clade D
5-aminolevulinate synthase, nonspecific, mitochondrial TRINITY_DN345597_c1_g1_i1
ALAS
Uroporphyrinogen decarboxylase TRINITY_DN332132_c0_g1_i1
UROD
Uroporphyrinogen decarboxylase TRINITY_DN332132_c0_g2_i1
PPOX
Protoporphyrinogen oxidase TRINITY_DN346552_c1_g3_i2
FECH
Ferrochelatase, mitochondrial TRINITY_DN340657_c1_g3_i1
Protoporphyrinogen oxidase TRINITY_DN346552_c1_g3_i2
PPOX15
Protoheme IX farnesyltransferase, mitochondrial TRINITY_DN346156_c0_g1_i1
Cytochrome c oxidase assembly protein COX15 homolog TRINITY_DN347275_c1_g1_i1
COX15
Heme oxygenase 1 TRINITY_DN339369_c4_g1_i1
HO-1
Cytochrome c-type heme lyase TRINITY_DN345553_c1_g1_i1
Cytochrome c-type heme lyase TRINITY_DN345553_c1_g7_i1
CCHL
Cytochrome c-type heme lyase TRINITY_DN345553_c1_g7_i4
Beta-hexosaminidase subunit alpha TRINITY_DN343301_c1_g6_i1
HEXA
Chitinase 1 TRINITY_DN335446_c0_g1_i2
CHIT
N-acetyl-D-glucosamine kinase TRINITY_DN330849_c0_g1_i2
Alpha-aminoadipic semialdehyde synthase, mitochondrial TRINITY_DN342261_c0_g1_i1
AASS
ALDH7A1
Alpha-aminoadipic semialdehyde dehydrogenase TRINITY_DN338204_c0_g1_i1
Histone acetyltransferase KAT2B TRINITY_DN337574_c0_g2_i1
DLD
Dihydrolipoyl dehydrogenase, mitochondrial TRINITY_DN338771_c1_g1_i2
Glutaryl-CoA dehydrogenase, mitochondrial TRINITY_DN346337_c1_g8_i2
GCDH
Glutaryl-CoA dehydrogenase, mitochondrial TRINITY_DN346337_c1_g8_i4
Ethylmalonyl-CoA decarboxylase TRINITY_DN343424_c2_g1_i2
ECHD
Ethylmalonyl-CoA decarboxylase TRINITY_DN343424_c2_g1_i4
Enoyl-CoA hydratase domain-containing protein 3, mitochondrial TRINITY_DN343172_c1_g2_i1
ECHS1
Acetyl-CoA acetyltransferase, mitochondrial TRINITY_DN346847_c1_g3_i1
ACAT
BCAT
Branched-chain-amino-acid aminotransferase, cytosolic TRINITY_DN344803_c1_g1_i1
BCKDHA
2-oxoisovalerate dehydrogenase subunit alpha, mitochondrial TRINITY_DN344194_c0_g5_i1
Dihydrolipoyl dehydrogenase, mitochondrial TRINITY_DN338771_c1_g1_i2
DLD
Nitric oxide synthase, endothelial TRINITY_DN268744_c0_g1_i1
Nitric oxide synthase, brain TRINITY_DN328766_c0_g1_i1
Nitric oxide synthase-interacting protein TRINITY_DN341142_c1_g1_i1
NOS
Nitric oxide synthase, brain TRINITY_DN346758_c0_g1_i1
PK
Pyruvate kinase PKM TRINITY_DN336206_c1_g1_i1
Pyruvate dehydrogenase E1 component subunit alpha, somatic form, mitochondrial TRINITY_DN319839_c0_g1_i2
PDCE2
Fatty acid synthase TRINITY_DN344489_c1_g1_i1
FASN
PK
3-ketoacyl-CoA thiolase, mitochondrial TRINITY_DN343517_c3_g1_i1
LCAD
Long-chain specific acyl-CoA dehydrogenase, mitochondrial TRINITY_DN340949_c0_g2_i1
Carnitine O-palmitoyltransferase 2, mitochondrial TRINITY_DN343414_c1_g1_i1
CPT
Palmitoyl-protein thioesterase 1 TRINITY_DN328632_c2_g1_i1
PPT1
Palmitoyl-protein thioesterase 1 TRINITY_DN328632_c2_g1_i3
Control
Day 1
Day 2
Control
Day 1
Day 2

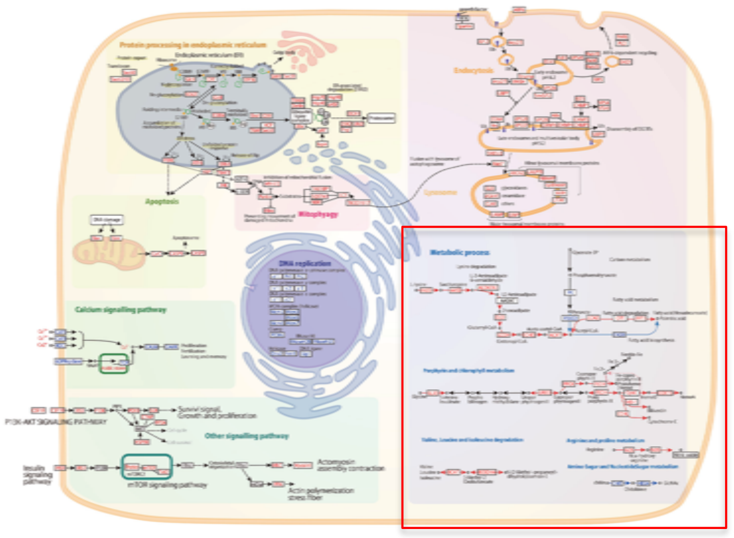

Figure S8. Expression pattern of algal DEG (differentially expressed genes) related to GO term in figure 6. Heat map representation of each gene expression pattern between control conditions and stress conditions: yellow highlight being higher expressed and blue highlight being lower expressed. Each expression values were converted into z-score and plotted by heatmap. 2 in R. Only major GO terms are listed. The abbreviations for the genes listed in 6 are shown in red near their gene expression data.

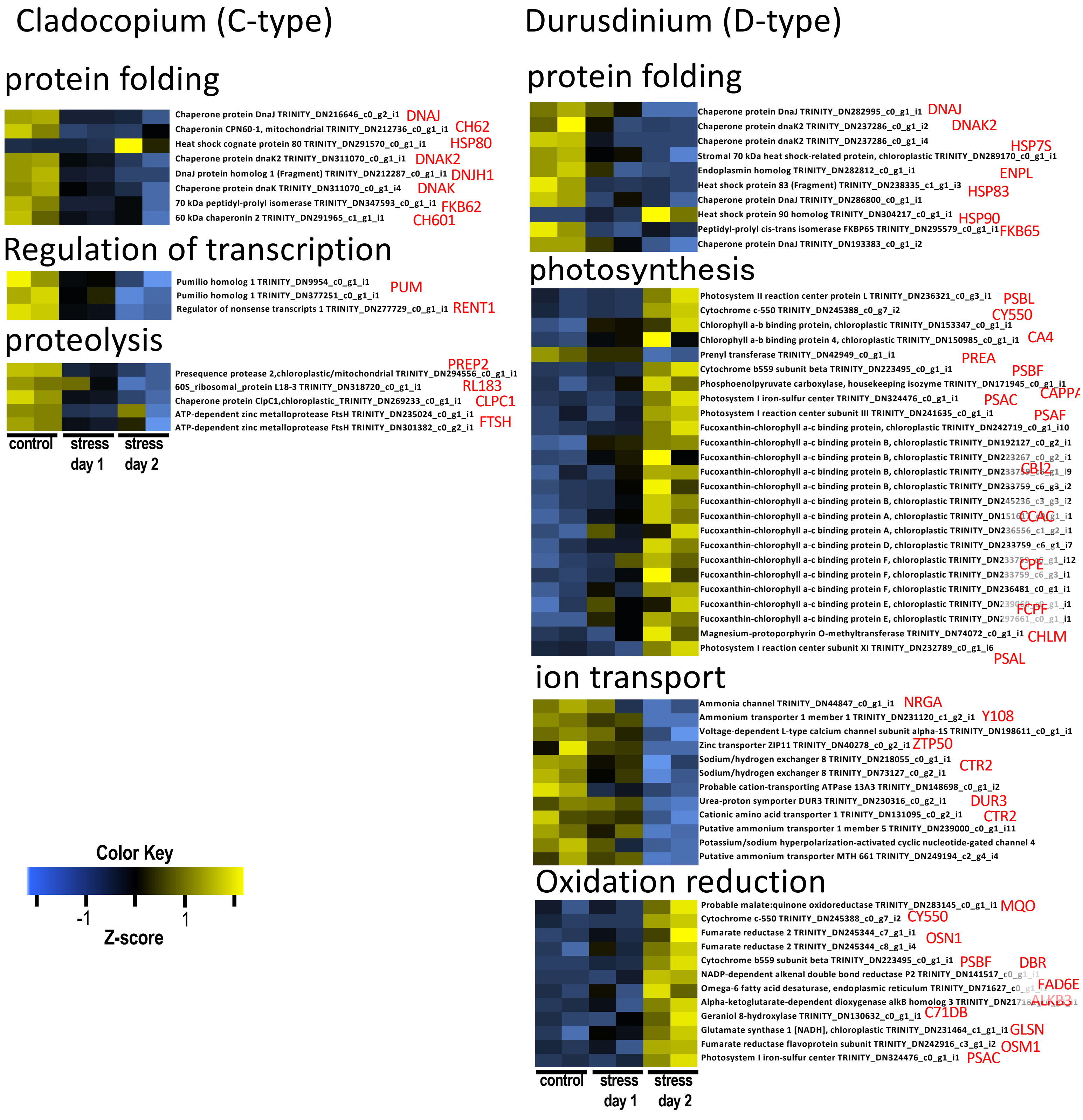

Table S1. The number of aligned sequences to the database.
Sequences aligned only with the coral database were designated as *A. solitaryensis* (coral) derived contigs (A). Sequences aligned only with the Symbiodiniaceae database were designated as Symbiodiniaceae (algal) derived contigs (B). The coral derived contigs and algal derived contigs were used as reference gene sets for differentially expression analysis.

(A)

	contigs from all samples
aligned with symbiodiniaceae sequences	45,209
aligned with coral sequences	41,343
aligned with both (coral and Symbiodiniaceae) sequences	1,307
aligned only with coral sequences	40,036

(B)

	contigs of Cladocopium-symbiosis	contigs of Durusedinium-symbiosis
aligned with symbiodiniaceae database	44,822	20,709
aligned with coral database	31,723	31,657
aligned with both (coral and Symbiodiniaceae) database	376	341
aligned only with symbiodiniaceae database	44,446	20,368

Table S2. Statistics of obtained Symbiodiniaceae (Cladocopium and Durusdinium) derived contigs and *Acropora solitaryensis* derived contigs

Statistic	Cladocopium derived contigs	Durusdinium derived contigs	*A. solitaryensis* derived contigs
Total length (bp)	51,905,796	30,472,320	35,974,968
Number of contigs	44,446	20,368	40,036
Average length	1,168	1,496	899
Median length	927	1,230	558
Max length	14,022	14,082	19,335
Min length	297	297	297
N50	1,509	1,836	1,221
GC content	55.00%	56.00%	43.92%
